# Modified influenza M1_58–66_ peptide vaccination induces non-relevant T-cells and may enhance pathology after challenge

**DOI:** 10.1038/s41541-023-00705-y

**Published:** 2023-08-12

**Authors:** Josien Lanfermeijer, Koen van de Ven, Harry van Dijken, Marion Hendriks, Cami M. P. Talavera Ormeño, Femke de Heij, Paul Roholl, José A. M. Borghans, Debbie van Baarle, Jørgen de Jonge

**Affiliations:** 1 Center for Infectious Disease Control, National Institute for Public Health and the Environment, Bilthoven, the Netherlands; 2 Center for Translational Immunology, University Medical Center Utrecht, Utrecht, the Netherlands; 3 Department of Cell and Chemical Biology, Leiden University Medical Centre, Leiden, The Netherlands; 4 Microscope Consultancy, Weesp, Netherlands; 5 Present Address: Virology & Immunology Research. Dept Medical Microbiology and Infection prevention, University Medical Center Groningen, Groningen, the Netherlands

**Keywords:** Peptide vaccines, Influenza virus, Cellular immunity

## Abstract

CD8 + T cells are promising targets for vaccination against influenza A virus (IAV) infection. Their induction via peptide vaccination is not trivial, because peptides are weakly immunogenic. One strategy to overcome this is by vaccination with chemically enhanced altered peptide ligands (CPLs), which have improved MHC-binding and immunogenicity. It remains unknown how peptide-modification affects the resulting immune response. We studied the effect of CPLs derived from the influenza M1_58–66_ epitope (GILGFVFTL) on the T-cell response. In HLA-A2*0201 transgenic mice, CPL-vaccination led to higher T-cell frequencies, but only a small percentage of the induced T cells recognized the GILG-wildtype (WT) peptide. CPL-vaccination resulted in a lower richness of the GILG-WT-specific T-cell repertoire and no improved protection against IAV-infection compared to GILG-WT peptide-vaccination. One CPL even appeared to enhance pathology after IAV-challenge. CPL-vaccination thus induces T cells not targeting the original peptide, which may lead to potential unwanted side effects.

## Introduction

Despite the availability of vaccines, influenza A virus (IAV) infection is still a worldwide health threat. Traditional influenza vaccines induce humoral immune responses against the highly variable viral surface proteins haemagglutinin (HA) and neuraminidase (NA)^1^, which are relatively narrow and therefore mainly strain-specific. Via mutations in these surface proteins (antigenic drift), IAV can escape previously induced immunity. In contrast to humoral immune responses, cellular immune responses are often directed against more conserved parts of the virus. Cellular immunity may therefore provide cross-protection against seasonal drifting strains and against newly emerging influenza viruses with pandemic potential. This is corroborated by the observation that individuals with pre-existing IAV-specific T-cell immunity have an immunological advantage resulting in (partial) protection upon encounter with a new IAV infection^2–4^. Therefore, induction of cellular responses against the more conserved parts of the virus is of great interest for the design of new, broadly-reactive vaccines against IAV.

One way to induce cellular responses is via peptide vaccination. Peptides alone, however, tend to be weak immunogens. To overcome this, different approaches to induce robust T-cell responses by peptide-based vaccination have been tested, including i) vaccination with long synthetic peptides to simultaneously activate CD8+ and CD4 + T-cells^5^, ii) the use of strong adjuvants or immune stimulants, as seen in peptide-conjugate vaccines^6^, and iii) optimization of the drug delivery system^6^. Previously, we conducted a study with chemically enhanced altered peptide ligands (CPLs) to induce IAV-specific T-cell responses^7^. By changing the residues of a peptide at or near the anchor into non-proteogenic amino acids, the binding affinity of the peptide to the MHC molecule can be increased, thereby prolonging the presentation of vaccine peptides on the cell surface. A prerequisite for the success of this approach is that most T cells induced by CPL-vaccination should be able to recognize the wildtype (WT) viral peptide. We hypothesized that prolonged presentation would lead to enhanced T-cell immunogenicity and a broader T-cell response, both at the clonal level (i.e. more T-cell receptor (TCR) diversity) and in terms of cross-reactivity against naturally occurring viral mutants, since T cells would have more time to recognize the peptide, allowing more clones to react. Furthermore, the incorporation of non-proteogenic amino acids may make CPLs resistant to proteolytic degradation. We indeed observed that several CPLs had enhanced MHC-binding affinity compared to natural IAV-peptides, and that vaccination of mice with CPLs induced a higher IFNγ-response against the WT peptide compared to vaccination with the WT peptide^7^.

In the current study, we investigated additional features of the T-cell response after vaccination with CPLs, including the frequencies of peptide-specific CD8 + T cells, and their IFNγ-expression, cross-recognition and TCR repertoire diversity. We focused on the response against the IAV-specific matrix protein 1_58–66_ (M1_58–66_) GILGFVFTL (GILG) epitope, which is highly immuno-dominant in humans with HLA-A2^8^. The GILG epitope is highly conserved between different IAV strains, emphasizing the advantage of inducing a robust response against this epitope^9^. We selected four different CPLs of the GILG epitope, all with enhanced binding affinity to the HLA-A2 molecule, but resulting in different T-cell responses after vaccination compared to vaccination with the WT peptide^7^. We evaluated the characteristics of the different responses that were induced in vivo, in a transgenic mouse model expressing a hybrid class I MHC gene, containing the alpha-1 and alpha-2 domain of the human HLA-A2.1 gene. We found that CPL-vaccination induced higher T-cell frequencies than vaccination with the WT peptide. The majority of the induced T cells, however, did not recognize the WT peptide, and are therefore considered non-relevant. Furthermore, vaccination with CPLs led to a more skewed T-cell repertoire against the WT peptide. Most importantly, we found that CPL-vaccination did not provide better protection against IAV-challenge than vaccination with the WT peptide. Vaccination with one of the CPLs even tended to cause enhanced pathology after IAV-challenge. These findings show that modified-peptide vaccine strategies may induce a substantial amount of non-relevant, and possibly even detrimental, T cells. Such strategies should thus be monitored carefully when used to protect against infectious diseases.

## Results

### Chemically altered peptides

In an earlier study, IAV-specific peptides were chemically altered near or at the MHC-anchor residues using non-proteogenic amino acids^7^. The chemical alterations were rationally designed based on available crystal structures of peptide-HLA-A2 complexes and on side chain similarities. This way the binding to HLA-A2 was enhanced, which in some cases resulted in a higher IFNγ-response upon CPL compared to WT-peptide vaccination in mice. In the current study, we selected four modifications (MOD1, MOD2, MOD3 and MOD4, see Table 1) of the GILGFVFTL peptide to acquire a deeper understanding of the effect of these modifications on the T-cell response after vaccination. These CPLs were selected based on their enhanced MHC-binding scores compared to the WT peptide (Table 1) and subsequent different outcomes in their enhancement of the IFNγ-response^7^.

**Table 1:** Overview of peptide modifications and corresponding binding affinity to MHC.

	Amino Acid sequence + modification	Original name^1^	Binding^2^ (% of inhibition) after 4 h	Binding^2^ (% of inhibition) after 24 h
**WT**	GILGFVFTL		85 ± 0	84 ± 2
**MOD1**	[am-phg]ILGFVFTL	G1	97 ± 4	98 ± 4
**MOD2**	[3-PYRA]ILGFVFTL	G8	94 ± 3	93 ± 1
**MOD3**	G[NLE]LGFVFTL	G16	91 ± 3	90 ± 5
**MOD4**	[SOME]ILGFVFTL	G25	89 ± 12	89 ± 13

^1^This data was previously published in the study of Rosendahl Huber et al. ^7^.

^**2**^HLA-A*0201 binding affinity was determined by a Fluorescence Polarization-Based Peptide Binding Assay^7^. Binding was scored as percentage inhibition of tracer peptide binding after 4 h and 24 h.

### Dose-response experiment MOD1

**Fig. 1:**
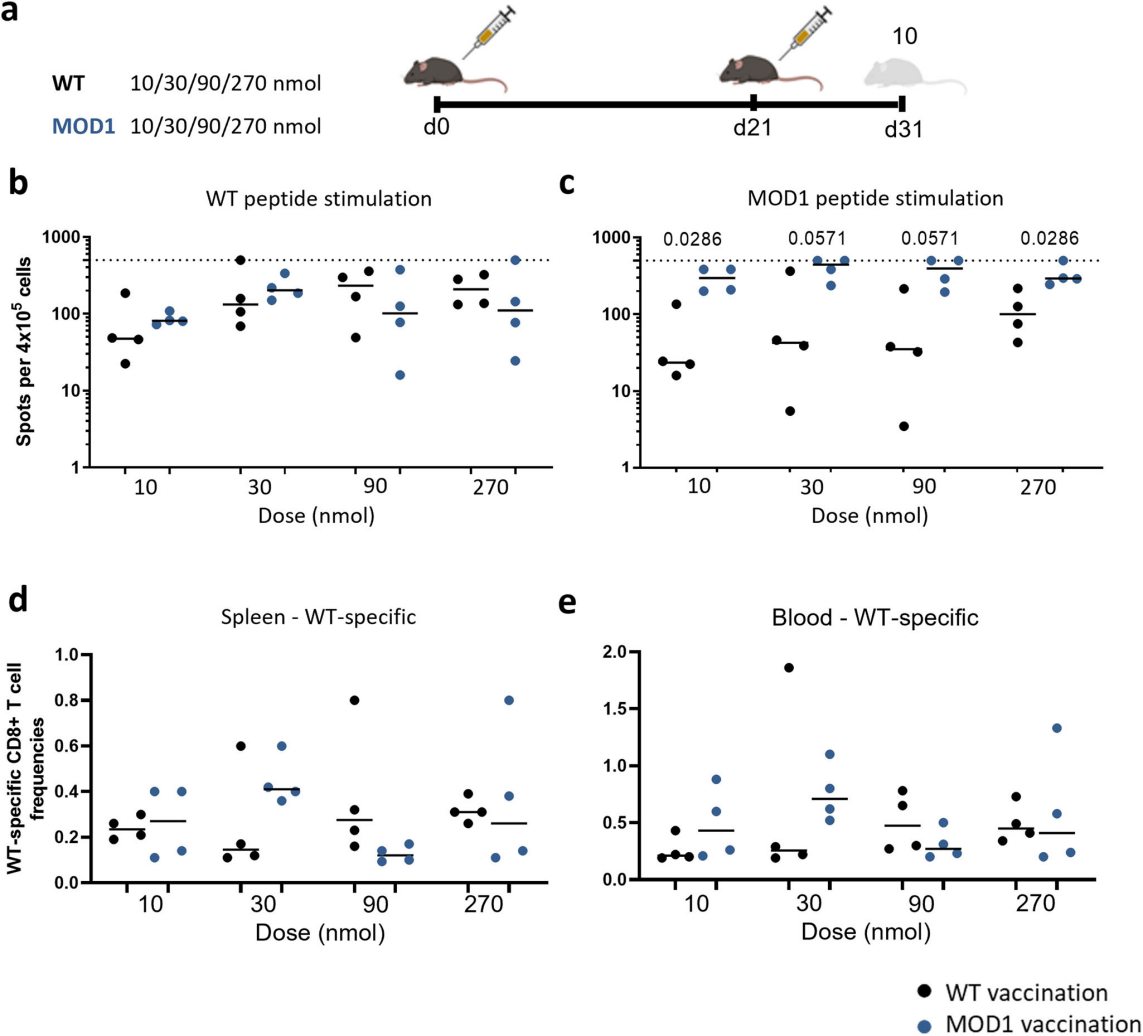
Dose-response determination with WT and MOD1 peptide vaccination. **a** Study layout depicting the prime-boost strategy. Mice were primed at day 0 by vaccination with either WT peptide or MOD1 peptide in a dose range of 10–30–90–270 nmol and received the same treatment 21 days later (*n* = 4 per treatment). The experiment was controlled by a mock vaccination (day 0 and day 21) and an influenza virus infection (day 21 only). Lymphocytes were isolated from spleen and blood 10 days post booster (dpd) vaccination. **b**, **c** Cellular responses were measured by IFNγ-ELISpot after vaccination. 4 × 10^5^ cells per condition were stimulated with (**b**) WT peptide or (**c**) MOD1 peptide. The horizontal dotted line depicts the upper limit of detection (500 spots). **d**, **e** WT-specific CD8 + T-cell frequencies were measured by dextramer staining in (**d**) spleen and (**e**) blood of mice vaccinated with WT or MOD1 peptide. In (**b**–**e**) results of individual mice are shown (points) with the group median (line). Mice vaccinated with WT peptide are depicted in black, whereas mice vaccinated with MOD1 peptide are depicted in dark blue. Differences between groups were tested for significance by one-way ANOVA, followed by a post-hoc Mann-Whitney U test. Only significant p-values or p-values depicting a trend are depicted in the graphs. Study layout was created using BioRender.com.

First we performed an extended dose-finding study with a broader range of peptide concentrations and more animals, to select a dose for further immunological analysis of the T-cell responses induced by the CPLs. To this end, we selected the WT peptide and one CPL (MOD1) that previously showed an improved response^7^. We prime-boosted HLA-A2 transgenic mice with either 10, 30, 90 or 270 nmol of the WT or MOD1 peptide, with an interval of 21 days between prime and boost (Fig. 1a).

Ten days post booster vaccination, we observed that the IFNγ-response of splenocytes induced by MOD1-vaccination already reached its maximum at 30 nmol in an ELISpot assay after ex vivo restimulation with WT peptide (Fig. 1b, controls are shown in Supplementary Fig. 1a). At higher doses, the response started to plateau. There was no significant difference in the response induced by WT-vaccination or MOD1-vaccination for any of the doses. Ex vivo stimulations with MOD1 peptide induced a very high response in MOD1-vaccinated mice and a lower response in mice vaccinated with WT peptide (Fig. 1c, controls are shown in Supplementary Fig. 1a).

When we analyzed the peptide-specific T-cell frequencies by staining with WT-loaded HLA-A2 dextramers, we observed a clear subset of WT-specific T cells in both WT and MOD1-vaccinated mice, in both spleen and blood (Fig. 1d, e, Supplementary Fig. 1c). Splenocytes isolated from mock-vaccinated mice and IAV-infected mice served as staining controls (Supplementary fig. 1b, c). Comparable to what we observed in the IFNγ-ELISpot assay, the highest response of the WT-specific T-cell frequencies was already reached at a vaccination dose of 30 nmol. Based on these results, we selected 30 nmol as the dose for an in-depth mechanistic analysis of the four selected CPLs.

### Dissecting the T-cell response induced by the different CPLs

**Fig. 2:**
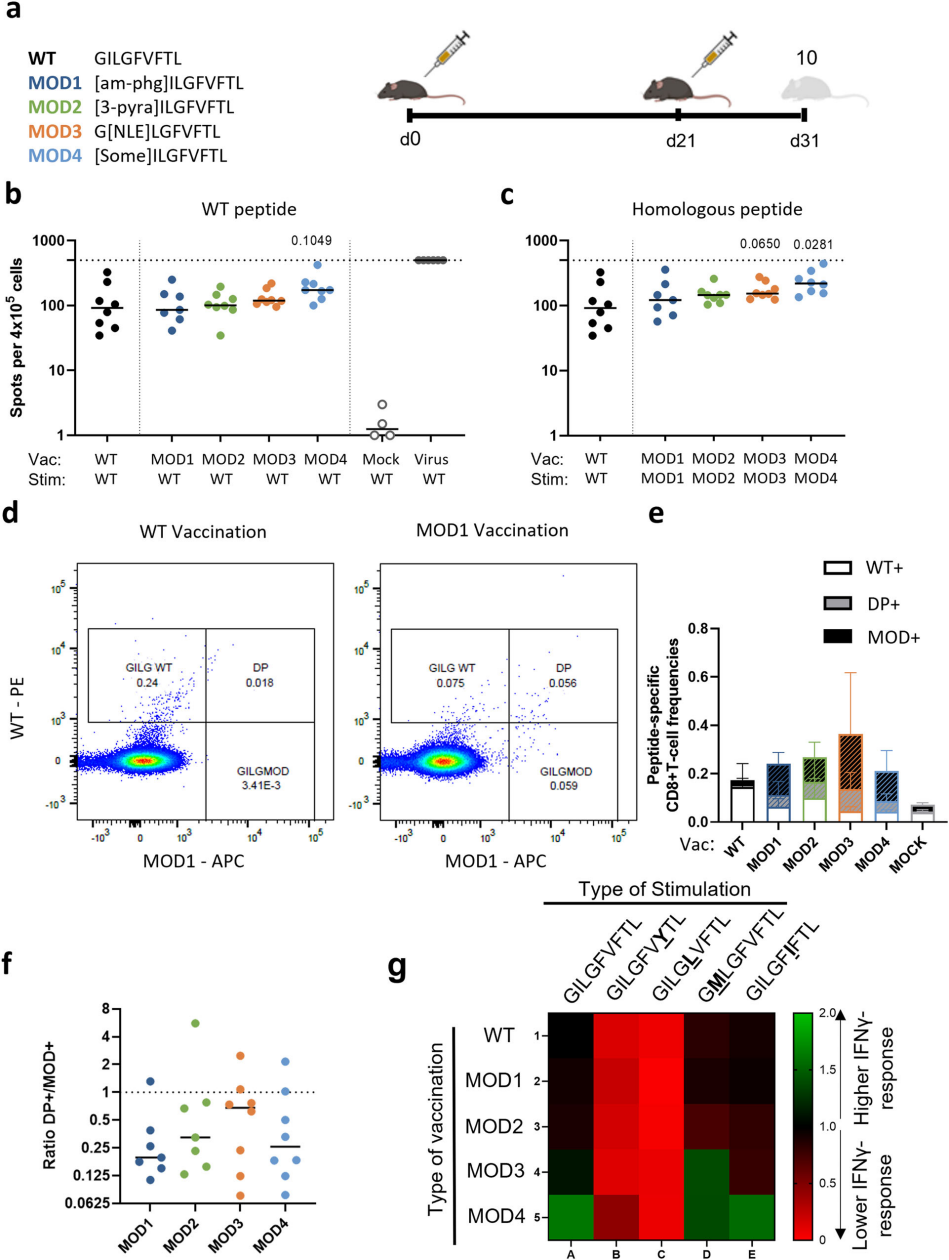
Vaccination with modified peptides induces non-relevant T-cell responses. **a** Study layout depicting the prime-boost strategy used to compare WT peptide vaccination with CPL vaccination (MOD1–4). Mice were primed with 30 nmol peptide vaccine (either WT or one of four CPLs) and were boosted 21 days later. 10 days post booster (dpd) mice were sacrificed and splenocytes were isolated. **b**, **c** Cellular responses were measured by IFNγ-ELISpot assay after restimulation of 4 × 10^5^ splenocytes with (**b**) WT peptide or (**c**) homologous restimulation (WT peptide or MOD1–4). The horizontal dotted line depicts the upper limit of detection (500 spots). **d** Example of gating strategy in mice vaccinated with WT (left) and MOD1 (right) peptide using two different dextramers, one loaded with WT peptide (PE) and one loaded with MOD1 peptide (APC). **e** Overview of peptide-specific T-cell frequencies present in the WT + , DP+ (double positive) and MOD+ gate. Slashes depict the specific T-cell populations that recognize the respective CPL (DP+ and MOD + ). **f** Ratio of number of T cells in the DP+ gate and number of T cells in the MOD+ gate after vaccination with one of the four CPLs. The higher the ratio, the larger the fraction of T cells recognizing both the WT and respective MOD peptide. **g** Heat-map of the IFNγ-response against 4 natural variants of the WT GILG peptide as measured with IFNγ-ELISpot. Naturally occurring mutations in the GILGFVFTL epitope are indicated in bold and underlined. Responses against WT by mice vaccinated with WT peptide were used as reference response and set to 1. The numbers indicate fold change compared to the reference response. Green indicates higher response compared to reference response, red indicates lower response compared to the reference response. **b**, **c**, **f** Results of individual mice are shown (points) with the group median (line). In (**e**), the mean ± SD per group is shown. Mice vaccinated with WT are depicted in black and mice vaccinated with CPL are indicated by the following colors: MOD1 – dark blue, MOD2 – green, MOD3 – orange, MOD4 – light blue. Differences between groups were tested for significance by one-way ANOVA, followed by a post-hoc Mann-Whitney U test. Only significant *p*-values or p-values (MOD vs WT) depicting a trend are depicted in the graphs. Study layout was created using BioRender.com.

To compare the magnitude of the T-cell responses induced by the different peptide modifications and the WT peptide, we prime-boosted mice with 30 nmol of WT or one of the four CPLs (*n* = 8 per treatment) (Table 1, Fig. 2a). Ten days after boosting, restimulation of splenocytes with the GILG-WT peptide in an IFNγ-ELISpot assay showed that responses of CPL-vaccinated mice were comparable to those of mice vaccinated with WT (Fig. 2b). When stimulating the splenocytes with the peptide that was used for the vaccination, the IFNγ-response to MOD4 was significantly higher than the response to the WT peptide (Fig. 2c). The responses to the other CLPs were not significantly different from the response to WT, although MOD3 showed a trend to a higher response after homologous stimulation. The response after vaccination with the WT-peptide or CPLs was much lower compared to the response after virus infection (Fig. 2b, c). Although based on the results of our previous study we expected vaccination with all four CPLs to induce a higher IFNγ-response to the WT peptide than vaccination with the WT peptide itself^7^, the current dataset (which has a higher power), shows that in fact vaccination with none of the modified peptides induces a significantly increased IFNγ-response to the WT peptide.

Next, we wondered if CPLs would also be able to boost a response induced by a previous influenza infection as this more reflects the human situation. To mimic this scenario, we stimulated splenocytes of mice infected with IAV with one of the four CPLs. The ELISpot results show that all four CPLs result in activation of an infection-induced response (Supplementary Fig. 2) indicating that the CPLs potentially boost memory response induced by a previous influenza infection.

To distinguish between T cells that recognize the WT peptide or only the CPLs, we combined a CPL- and WT peptide loaded dextramer staining to distinguish between CD8 + T cells recognizing only the WT peptide (WT + ), only the CPL (MOD + ) or both (Double Positive, DP + ) (Fig. 2d, Supplementary Fig. 3A). WT-vaccinated mice, had a population of WT + T cells, as well as a DP + T-cell population, recognizing both the WT peptide and the CPL (Fig. 2d, e). Mice vaccinated with CPLs had both MOD+ and DP + T-cell populations.

To investigate whether CPL-vaccination in general resulted in a higher T-cell response, we compared the total dextramer response (sum of WT + , DP+ and MOD + ) induced by the different vaccinations. Vaccination with MOD1, 2 and 4 did not induce higher total T-cell frequencies compared to vaccination with WT peptide (Fig. 2e). The total dextramer+ T-cell response induced by MOD3-vaccination was significantly higher than the response induced by WT-vaccination (*p* = 0.0499, Supplementary Fig. 3B). In line with the IFNγ-responses, T-cell frequencies measured by dextramer staining were significantly lower after peptide vaccination than after virus infection, both in terms of the WT-specific response and the total dextramer+ T-cell response (Supplementary Fig. 3B). Importantly, for all four CPL vaccinations, we observed that the induced T-cell response consisted of a relatively larger fraction of T cells that only recognized the specific CPL, both in spleen (Fig. 2e) and in the blood (Supplementary Fig. 3B). T cells recognizing only the CPLs and not the WT peptide are unlikely to contribute to T-cell protection upon IAV infection and are thus presumably non-relevant. To determine to which extent CPLs induced useful T cells, we calculated the ratio between the frequency of T cells recognizing both the WT peptide and the CPL (DP + ) and the frequency of T cells recognizing only the CPL (MOD + ). Although there was a large variation within groups, the amount of T cells recognizing the WT peptide was between 2 to 5 times smaller than the amount of T cells recognizing the CPL (Fig. 2f). Thus, although vaccination with some of the CPLs resulted in a higher IFNγ-response to the CPL and higher T-cell frequencies compared to WT-vaccination, the responses induced by CPLs consisted to a large extent of non-relevant T cells.

### Recognition of naturally occurring peptide variants

Although the WT peptide is known to be relatively conserved, natural variants do occur. We wondered if CPL-vaccination could enhance the recognition of these natural variants when compared to vaccination with WT. We selected four different naturally occurring peptide variants for ex vivo restimulation, namely GILGFV**Y**TL, GILG**L**VFTL, G**M**LGFVFTL and GILGF**I**FTL, based on the highest frequency of occurrence^10^. T cells induced by vaccination with WT showed a comparable IFNγ-response against two other natural variants, G**M**LGFVFTL and GILGF**I**FTL, whereas GILGFV**Y**TL and GILG**L**VFTL were recognized poorly (Fig. 2g, Supplementary Fig. 4). T cells induced by vaccination with any of the four CPLs also recognized GILGFV**Y**TL and GILG**L**VFTL relatively poorly. On the other hand, MOD3-vaccination resulted in higher responses against G**M**LGFVFT (*p* = 0.0350) compared to WT-vaccination, whereas vaccination with MOD4 resulted in higher responses against GILGF**I**FTL after restimulation (*p* = 0.0499) in comparison with WT-vaccination (Supplementary fFg. 4). This suggests that some CPLs may enhance the recognition of naturally occurring GILGFVFTL viral variants.

### Effect of peptide modification on the TCR repertoire of WT-specific T cells

**Fig. 3:**
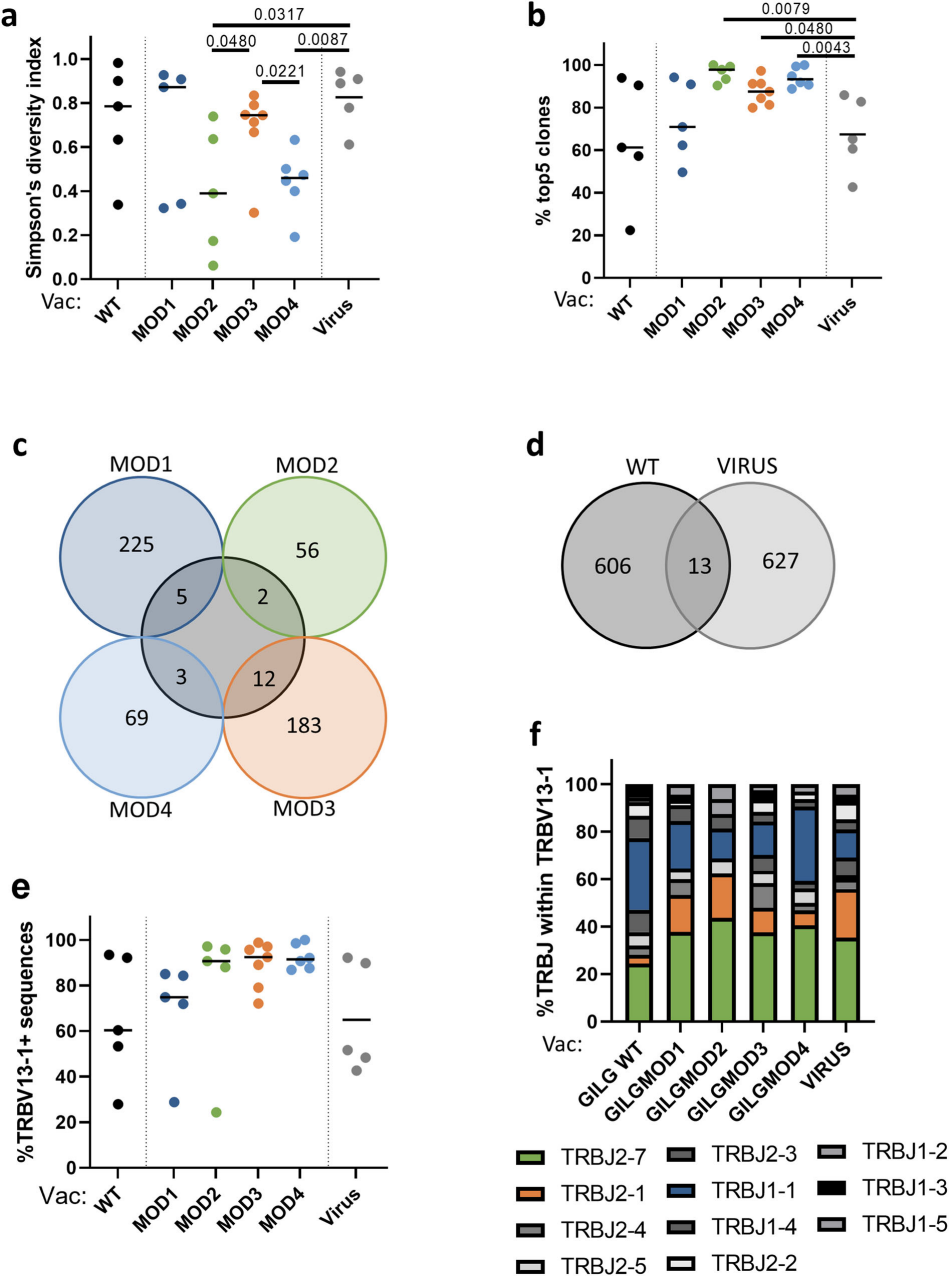
GILG-WT specific T-cell repertoire analyses after peptide vaccination. **a** Simpson’s diversity index of the WT-specific TCR repertoire after WT or CPL vaccination and influenza virus infection. **b** Skewing of the WT-specific TCR repertoire was calculated by the contribution of the top 5 largest clones per sample in percentages. **c** Overview of the number of shared sequences between mice vaccinated with WT or one of the four CPLs. TCR sequences were merged per group. **d** Overview of the number of GILG WT-specific TCR sequences shared between mice vaccinated with WT peptide or infected with influenza virus (PR8). **e** Overview of the percentage of sequences containing TRBV13–1. **f** Overview of the different JB segments withing the VB13+ sequences, TCR sequences were merged per group. **a**, **b**, **e** Results of individual mice are shown (points) with the group median (line). Mice vaccinated with WT peptide are depicted in black and mice vaccinated with CPL are indicated by the following colors: MOD1 – dark blue, MOD2 – green, MOD3 – orange, MOD4 – light blue. Mice infected with influenza a virus are shown as grey colored closed circles. Differences between responses to WT and CPLs are tested using Mann-Whitney U test. *P*-values are only shown when there was a significant difference between WT and a MOD vaccination.

We hypothesized that vaccination with CPLs would lead to a broader TCR repertoire diversity of WT-specific T cells, as extended peptide presentation allows for recognition by more T-cell clones. To investigate this, we sorted T cells recognizing the WT peptide and used Vβ sequencing to determine the WT specific TCR repertoire. The diversity of the TCR repertoire was calculated using the Simpson Diversity Index^11^. We observed relatively large within-group variation in the TCR diversity of the WT-specific T-cell pool, and no significant differences between mice vaccinated with WT peptide or with any of the four CPLs. However, the diversity of the TCR repertoire induced by MOD2 and MOD4-vaccination were both significantly lower compared to the TCR diversity induced by IAV infection (Fig. 3a). This was due to the presence of clonal expansions in MOD2 and MOD4-vaccinated mice, as the top five T-cell clones in these mice covered a significantly larger fraction of the induced T-cell pool than in IAV-infected mice (Fig. 3b). This difference was confirmed by a significantly lower TCR richness in MOD2 and MOD4 vaccinated mice when compared to WT-vaccinated mice and mice infected with IAV (Supplementary Fig. 5A). The diversity of the WT-specific repertoire induced by WT-vaccination was very similar to the repertoire after natural infection, despite the large difference in WT specific T-cell frequencies. The similarity was reflected in a comparable TCR repertoire diversity, evenness and richness (Fig. 3a, b, Supplementary Fig. 6A).

It has previously been shown that modifications in peptides can alter the 3D structure of peptide-MHC complexes, leading to the binding of different T-cell receptors^12^. Therefore, we investigated the overlap in sequences and characteristics of the TCR repertoires induced by WT and CPL-vaccination. There was not much overlap in TCR sequences between WT-vaccinated and CPL-vaccinated mice (Fig. 3c). Although at first sight this seems to suggest that CPL-vaccination induces a completely different TCR repertoire, in fact also the number of shared sequences between different WT-vaccinated mice (4 shared sequences) and between WT-vaccinated and IAV-infected mice (13, Fig. 3d) was low. Vaccination with MOD3 led to the highest number of shared TCR sequences with WT-vaccination (Fig. 3c).

Focusing on other characteristics of the TCR repertoire, like V and J usage and CDR3 length of the sequences, showed a skewing towards the usage of Vβ13–1 in the TCR repertoire of WT-vaccinated and IAV-infected mice. This bias seemed even more pronounced in mice vaccinated with MOD2, MOD3 and MOD4 (Fig. 3e), although these shifts were not significant. Within the Vβ13–1+ sequences, we observed skewing towards the use of the Jβ segments 2–7, 2–1 and 1–1 for all peptide vaccinations, as well as after IAV infection (Fig. 3f). It has previously been proposed that TCRs with a relatively long CDR3 region are more flexible and thereby more cross-reactive than TCRs with short CDR3 regions^13^. The TCR repertoire after WT-vaccination showed a bias towards a CDR3 length of 9 or 10 amino acids, while the WT-specific repertoires induced by CPLs showed a more prominent skewing towards one length (8 amino acids for MOD1 and MOD2, 10 amino acids for MOD3 and 9 amino acids for MOD4, Supplementary Fig. 5B). Although MOD3 and MOD4 both induced higher IFNγ responses against some of the naturally occurring GILG variants than WT-vaccination (Fig. 2g), this was thus not associated with longer CDR3 regions of the responding T cells.

### T-cell response prior to and after IAV-challenge in peptide-vaccinated mice

**Fig. 4:**
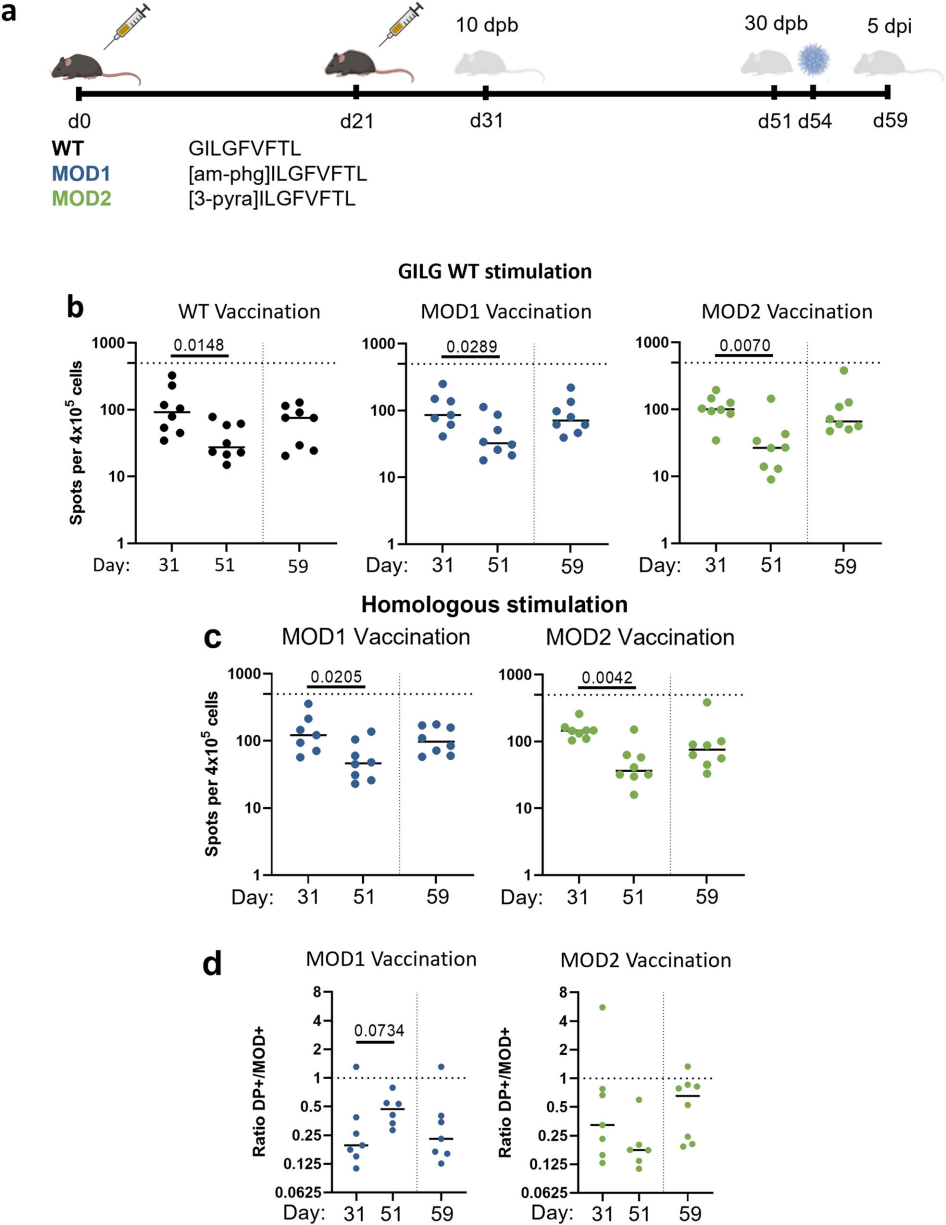
Memory response induced by peptide vaccination. **a** Study layout depicting the prime-boost strategy and subsequent IAV-challenge to compare the protective effect of WT vaccination with MOD1 or MOD2 vaccination. Mice were primed at day 0 with peptide vaccination (30 nmol) and received the same treatment 21 days later. At day 51 (30 days post booster (dpb)) half of the mice were sacrificed to study the memory T-cell responses. The other half received an IAV-challenge (PR8, 1 × 10^3^ TCID_50_) at day 54 (33 dpb) and were sacrificed 5 days post infection (dpi, day 59). **b**, **c** Cellular responses were measured by IFNγ-ELISpot after stimulation of 4 × 10^5^ splenocytes with (**b**) WT peptide or (**c**) homologous restimulation with MOD1 or MOD2. Horizontal dotted lines depict the upper limit of detection (500 spots). **d** Ratio of numbers of T cells in the DP+ and MOD+ gates after vaccination with MOD1 or MOD2, based on dextramer staining of CD8 + T cells. The lower the ratio, the larger the fraction of T cells recognizing only the MOD peptide. **b**–**d** Results of individual mice are shown (points) with the group median (line). Mice vaccinated with WT peptide are depicted in black. Mice vaccinated with CPL are indicated by the following colors: MOD1 – dark blue, MOD2 – green. Vertical dotted lines depict the timing of the IAV-challenge. Differences between groups were tested for significance by one-way ANOVA, followed by a post-hoc Mann-Whitney U test. *P*-values are only shown when there was a significant difference between WT and a MOD vaccination. Study layout was created using BioRender.com.

We next investigated the protective capacity of CPL-induced immunity in the early memory phase. We focused on MOD1 and MOD2-vaccination, as they were suggested to improve the T-cell response most strongly in our previous study^7^. For this purpose, we vaccinated mice with either WT, MOD1 or MOD2 peptide following the same prime-boost schedule as described earlier (Fig. 1) and subsequently challenged mice with the IAV PR8 strain, which contains the WT GILGFVFTL epitope, at day 54 (i.e., 33 dpb) (Fig. 4a). Half of the mice were sacrificed prior to IAV-challenge (day 51, 30 dpb) to study the early memory response present at the time of challenge. The other half of the mice were sacrificed 5 days post infection (dpi), i.e. at day 59, to analyze the immune response and the level of protection against IAV-challenge (Fig. 4a).

For all three peptide vaccinations, a clear contraction of the T-cell response was observed between day 31 and day 51 post booster vaccination, as measured by IFNγ-ELISpot after WT and homologous stimulation (Fig. 4b, c). The contraction of the response was similar for mice vaccinated with WT, MOD1 or MOD2. Dextramer staining showed that also after contraction of the response, a large fraction of the T cells induced by vaccination with CPLs only recognized the modified peptide (Fig. 4d).For all three peptide vaccinations, the IFNγ-response against WT was higher post IAV-challenge (day 59) than before challenge (day 51, Fig. 4b). This was also observed after homologous restimulation of T cells from CPL-vaccinated mice (Fig. 4c). The IFNγ-response in mock-vaccinated mice also increased after IAV-challenge (Supplementary Fig. 6B), but remained significantly lower than in WT and CPL-vaccinated mice, suggesting that the response in the vaccinated mice was a true memory response (Supplementary Fig. 6C). Next we calculated the ratio of T cells recognizing both peptides (DP + ) and cells recognizing only the modified peptide (MOD + ) (Fig. 4d, Supplementary Fig. 6A). Remarkably, the percentage of T cells recognizing the WT peptide seemed to increase after infection, however this was not significant (*p* = 0.0734, Fig. 4d). Unexpectedly, the fraction of T cells recognizing only the modified peptide increased after IAV-challenge of mice vaccinated with MOD1. This pattern was not observed in mice vaccinated with MOD2; in the latter mice, the ratio DP + /MOD+ cells remained close to one before and after IAV challenge.

### Clinical disease and pathology after IAV-challenge

**Fig. 5:**
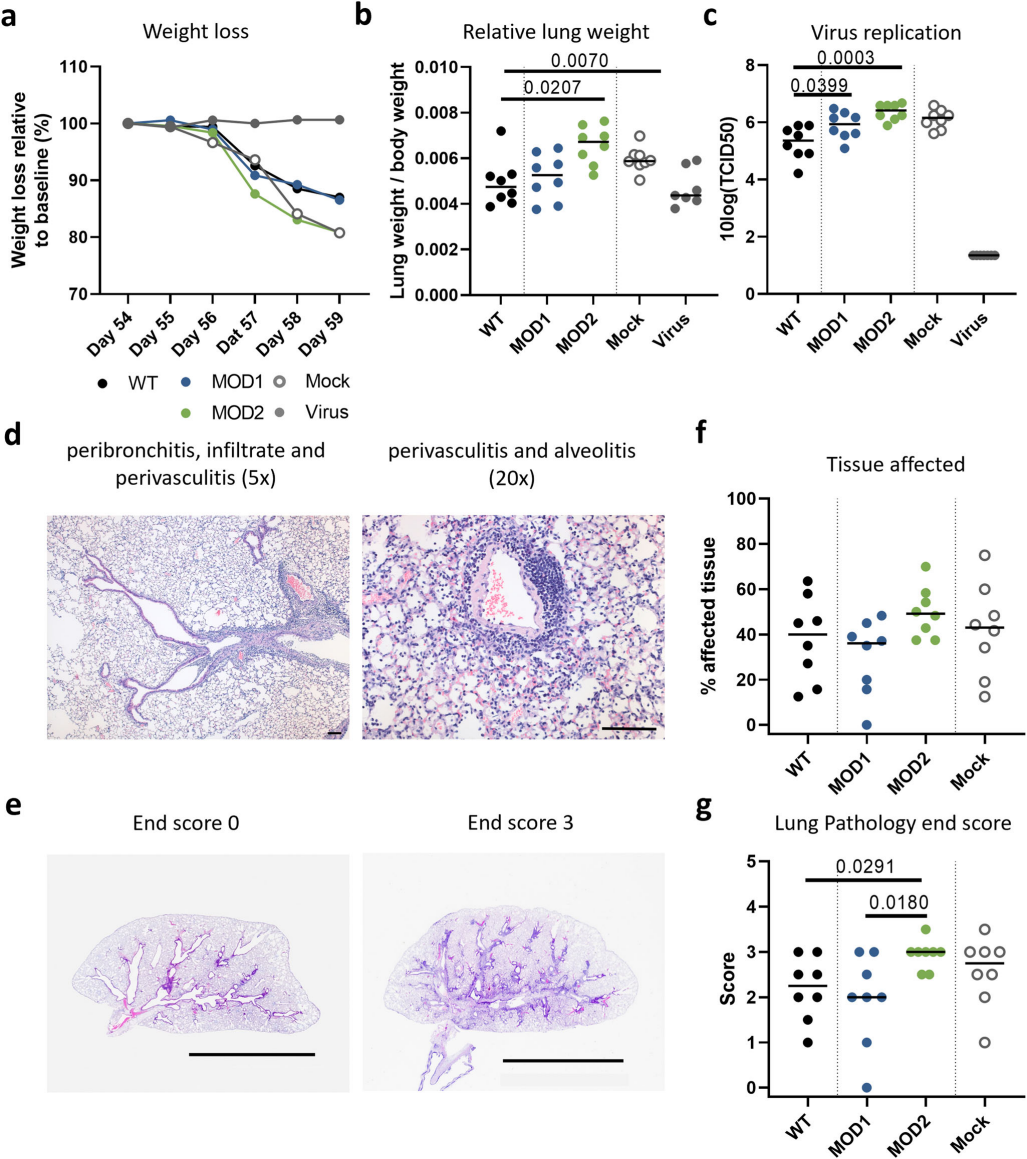
Clinical disease and pathology after IAV-challenge in peptide-vaccinated mice. Mice challenged with PR8 were sacrificed 5 days post infection (dpi) to determine pathology. **a** Weight loss in percentage after influenza infection at day 54. Weight at 54 days post booster (dpb; day of infection) was set at 100%. Mean per group is shown. **b** Lung/weight ratio, calculated by the weight of the right half of the lung at 59 dpd, divided by the total body weight at 54 dpb. **c** Virus titers in the left part of the lung at 59 dpd. **d** Illustrations of the histopathology of the lung. Histopathology was performed using H&E staining. Left image: Peribronchitis and infiltrate in the larger (not the smaller) bronchus and perivasculitis (5x magnification). Right image: Illustration of perivasculitis (score 3) and alveolitis (score 3, 20x magnification), bar 100 µm. **e** Overview of the histopathology of lungs with an end score of 0 (left image) and 3 (right image) illustrating damage and inflammation along the bronchi and bronchioli. Bar represents 0.5 cm. **f** Percentage of lung-tissue affected per mice. **g** End score (summarizing all the pathology scores) of the total histological damage based on scoring of all subcategories. Pathology was scored on a scale of 0 to 5. Per mouse at least 8 microscopic field were scored, median score per mouse was used. **b**, **c**, **f**, **g** Results of individual mice are shown (points) with the group median (line). Mice vaccinated with WT peptide are depicted in black. Mice vaccinated with CPL are indicated by the following colors: MOD1 – dark blue, MOD2 – green. Mock vaccinated mice are depicted in grey (open), IAV-infected mice are depicted in grey (solid). Differences between responses to WT and CPLs are tested using Mann-Whitney U test. *P*-values are only shown when there was a significant difference between WT and a MOD vaccination.

To assess whether peptide vaccination contributed to lower disease severity after IAV-challenge, i.e. whether it was associated with protection, we assessed clinical disease, pathology and virus replication after IAV-challenge in peptide-vaccinated mice. Mice vaccinated with WT or MOD1 showed a comparable relative weight loss after infection, which seemed a bit less than in mock-vaccinated mice (Fig. 5a). This might hint at some protection induced by WT and MOD1-vaccination, whereas the positive control mice, previously immunized with a virus infection, were completely protected. In contrast, MOD2-vaccinated mice showed the same relative weight loss as mock-vaccinated mice. An increase in relative lung weight (RLW, the ratio of the lung weight at termination to body weight at the day of challenge) resulting from edema and infiltrating leukocytes, is known to be a marker of disease in influenza infection^14^. Mice vaccinated with WT or MOD1 showed a comparable RLW at 5 dpi (Fig. 5b). The RLW in mice vaccinated with MOD2 and mock-vaccinated mice was significantly higher than in mice vaccinated with WT peptide. Surprisingly, the RLW of MOD2 mice appeared higher than mock-vaccinated mice although this was not significant. Virus titers were also significantly higher in the lungs of mice vaccinated with MOD2 than in mice vaccinated with WT peptide (*p* = 0.0003, Fig. 5c) and again appeared higher than in mock-vaccinated mice, but the difference was not significant. Although mice vaccinated with MOD1 showed a comparable relative weight loss and RLW as mice vaccinated with WT (Fig. 5b), a significantly higher virus titer was observed in the lungs of MOD1-vaccinated mice (*p* = 0.0399). Virus titers were absent and the RLW was not increased in the positive control. Together this suggests that vaccination with the CPLs investigated does not lead to better protection than vaccination with WT peptide and may even induce enhanced disease.

Next we assessed whether peptide vaccination could protect against lung pathology at the microscopic level. IAV Infection resulted in a mild to strong (peri) bronchitis, slight to mild (peri) bronchiolitis, slight to mild vasculitis and alveolitis in mice vaccinated with WT, MOD1, MOD2 or mock (Illustrated in Fig. 5d, e), however with different scores per treatment (Supplementary Fig. 7). All mice were scored based on the pathology in different parts of the lungs, e.g. bronchi, bronchioli, blood vessels, interstitium and alveoli (Fig. 5d). We found no significant differences between the different peptide vaccinations or mock-vaccination, in the percentage of affected tissue in the lungs (Fig. 5f). However, the end score (summarizing all the pathology scores) showed a significantly higher score (comparable to mock-vaccination) for the mice vaccinated with MOD2 compared to mice vaccinated with WT or MOD1 (Fig. 5g). This was primarily due to increased damage and inflammation in the bronchioli, two parameters very characteristic for influenza induced-lung pathology (Supplementary Fig 7). These parameters were even significantly higher in MOD2-vaccinated mice than in mock-vaccinated mice, which support the previous observed trends of an increased RLW and virus replication in MOD2 compared to mock-vaccinated mice (Supplementary Fig. 7). Surprisingly, WT and MOD1-vaccination did not lead to a significantly decreased pathology in the lungs compared to mock-vaccinated mice, although some parameters were reduced (i.e. alveolitis in WT peptide mice) (Supplementary Fig. 7). Together these data suggest that peptide vaccination -whether with CPLs or with WT peptide- does not lead to significantly enhanced protection against IAV infection. Mice vaccinated with MOD2 even showed increased pathology in the bronchioli after viral challenge compared to mock-vaccinated mice.

## Discussion

We investigated how vaccination with CPLs of the immunodominant conserved influenza GILG epitope influences the induced antigen-specific T-cell response in comparison to vaccination with the WT peptide. Although all four CPLs we used were previously shown to have increased binding affinity to the HLA-A2 complex^7^, we here show that vaccination with none of these CPLs leads to a larger T-cell response to the WT peptide. In fact, the majority of the induced T cells in CPL-vaccinated mice only recognized the CPL and not the WT peptide. Nevertheless, some of the CPLs showed enhanced recognition of naturally occurring viral GILGFVFTL variants. Furthermore, the T-cell repertoire directed against the WT peptide showed larger clonal expansions of only a few clones when induced by MOD2 or MOD4-vaccination compared to WT-vaccination. CPL vaccination showed a comparable contraction of the T-cell response in the memory phase as WT-vaccination, and did not lead to improved protection against IAV infection when compared to WT-vaccination. Vaccination with one of the CPLs even seemed to result in enhanced disease after IAV infection. Thus, CPLs induced a large number of “non-relevant” T cells, which failed to recognize the WT peptide, which might play a role in the enhanced pathology observed after IAV-challenge.

In this study, we chose to vaccinate with MHC-binding enhanced versions of the immuno-dominant GILGFVFTL peptide. This peptide seemed an ideal vaccine candidate as the GILG peptide is very conserved between IAV strains, most likely because mutations in the peptide lead to functional constraints for the virus^15^. The fact that almost all HLA-A2+ individuals have T cells specific for this peptide suggests the importance of this peptide in the CD8 + T-cell response against IAV infection. Also in HLA-A2 transgenic mice, the response against GILG was found to be dominant^16^. Here, we nevertheless found that vaccination with CPLs of GILG provided minor to no improvement in the cellular response against the GILG WT peptide. An explanation could be that it is harder to improve an already immunodominant response than a subdominant response. On the other hand, the responses induced by all peptide vaccinations were nowhere near the magnitude of the response induced by viral infection, suggesting that there should still be room for improvement. Nevertheless, we found that vaccination with WT and MOD1 provided better protection against IAV-challenge than mock-vaccination, based on weight loss and the relative lung weight. Thus, our data suggest that vaccination with a single peptide (WT or MOD1) could already lead to some level of protection. On the other hand, we also observed a significant increase in damage and inflammation of the bronchioli in MOD2-vaccinated mice compared to mock-vaccinated mice. These aberrations are typical for an influenza infection and their enhancement supports the increased trend in the relative lung weight, which is a non-biased measurement of cellular infiltration and edema formation. Altogether, this indicates that vaccination with CPLs can also lead to enhanced pathology after infection and that this aspects should be carefully considered when peptide vaccine strategies are further pursued.

It could be that vaccination with CPLs of subdominant peptides is in fact a more promising path. In line with this, we previously observed that the IFNγ-responses against the subdominant FMYSDFHFI and NMLSTVLGV peptides were improved more significantly by the introduction of non-proteogenic modifications than the response to GILG^7^. However, as the T-cell responses induced by WT FMYSDFHFI and WT NMLSTVLGV vaccination were low or non-detectable, modifications of these peptides were less suited for in depth immunological analysis, for which comparisons between WT and CPL induced T-cell responses are required. It would nevertheless be interesting to investigate the potential effect of vaccination with a combination of CPLs, targeting different peptides of the virus, as this may lead to a broader T-cell response^17^, more comparable to the response induced by virus infection. If these different peptides would also be presented on different HLA-types, a broader part of the population could be protected than only the HLA-A2+ individuals.

All CPLs that were investigated were modified at, or close to, the anchor residues of the GILGFVFTL peptide, which are important for binding to the MHC molecule (P1 or P2), with the underlying idea that the exposed part of the peptide that is recognized by the T-cell receptor would be left intact. We therefore expected that most T cells induced by CPL-vaccination would still be able to recognize the WT peptide. This was not the case, however. Based on dextramer staining we found that for all four CPLs, the majority of the induced T cells recognized only the modified peptide and not the WT peptide. We speculate that this may be due to a different positioning of the peptide in the HLA complex, resulting in a distinct presentation of the central part of the peptide. This phenomenon has indeed been shown for other peptides, for which one substitution at an anchor residue dramatically altered the conformation of the peptide-MHC complex^12^ or the conformation of the TCR contact sides^18^. It remains to be investigated whether the induction of a large fraction of non-relevant T cells is characteristic for the GILG peptide or whether it also occurs when modifying other influenza epitopes. It has been suggested that the GILG peptide is a featureless “plain vanilla” peptide, meaning that it lacks prominent side chains pointing towards the T-cell receptor when presented by HLA-A2^19,20^. Therefore, there may be fewer ways to bind the peptide and thus only a few TCRs may be sufficiently suited to bind the peptide-MHC complex. This may lead to a highly conserved and skewed T-cell repertoire recognizing this peptide-MHC complex in humans^21–23^. Therefore it could be possible that modifying the GILG peptide has even greater effects, as it may disrupt the “plain vanilla” binding, resulting in the possibility of more different TCRs to recognize the peptide-MHC complex, compared to peptides that are already presented in a different way.

All four CPLs investigated here induced a large fraction of non-relevant T cells that did not recognize the WT epitope. It would be interesting to further investigate the consequences of the induction of these non-relevant T cells and to study whether they play a role in the observed increased lung pathology in mice vaccinated with MOD2. It has recently been suggested that chemical modification directly alters the immunogenicity of a peptide and could thereby lead to the activation of potentially autoreactive T cells via molecular mimicry of endogenous ligands^24^. Whether this also played a role in the increased lung pathology observed in MOD2-vaccinated mice remains to be investigated.

To understand if a single amino acid substitution leading to enhanced HLA binding can influence the T cells recognizing the peptide, we also performed T-cell repertoire analyses. If anything, our data suggest that the enhanced MHC binding affinity of the tested CPLs led to more skewing of the repertoire towards Vβ13–1, and not to a broader repertoire as we hypothesized. Although one approach to enhance protection could be the selection of a superior T-cell clone^25^, this may not be preferable in the protection against IAV. It is generally thought that a broader TCR repertoire is preferable in protection against mutating viruses, as it increases the chance that T cells are present that can also recognize escape variants of the WT peptide, as has been observed for several other viral infections^26–28^. In this light, the increased skewing of the T-cell repertoire against the WT peptide that we observed after vaccination with some of the CPLs may not be preferable.

The highly skewed and public GILG WT-specific TCR repertoire in humans mostly consists of TCRs expressing TRBV19, TRAV27 and an RS motif in the CDR3β region^21,29^. In our mouse model, we also observed a strong skewing in the Vβ sequences, however towards TRBV13–1 (homologous to human TRBV10) and not towards TRBV19. We did not observe a dominant motif in the CDR3 regions of the TRBV13–1 + T-cell sequences. The mouse-model we used consists of a transgenic HLA-A2 molecule of which the alpha-3-domain is mouse-specific. This transgenic HLA-A2 molecule could play a role in the differences observed, as also the contact points of the MHC-molecule may play an important role in the TRBV19 skewing observed in humans.

After the IAV-challenge, we expected to see a shift towards the recognition of the WT peptide. Very much to our surprise, in mice vaccinated with MOD1, the fraction of T cells recognizing the WT peptide (useful T cells) decreased. As we only measured these frequencies in the spleen, it is tempting to speculate that in mice vaccinated with MOD1, the T cells that specifically recognized the WT peptide were present at the site of infection. This would be in line with the lower pathology observed in the MOD1-vaccinated mice. Further research on the presence of antigen-specific T cells in the lung would be needed to clarify this.

As influenza A virus infection remains a worldwide health threat, the development of new vaccination strategies is essential. There is a clear need for a more universal vaccine inducing protection against the ever-changing seasonal and potentially pandemic influenza viruses. A strategy that efficiently induces a cellular response against conserved epitopes holds great promise. Although peptide-based vaccinations have the potential to fill this gap, we show that the strategy to enhance MHC-binding by chemically altering the peptides to improve the immune response also has its limitations. More research is needed into different aspects of peptide vaccination in the battle against infections, including defining the right target-peptide, determining the effect on the diversity of the induced TCR repertoire and monitoring the induction of non-relevant T cells as an off-target immune response with possible unwanted side effects.

## Methods

### Ethical statement

The study was approved by the Animal Welfare Body of Poonawalla Science Park, Animal Research Center (Bilthoven, The Netherlands) under permit number AVD3260020173890 of the Dutch Central Committee for Animal experiments. All procedures were carried out in accordance with EU legislation. Mice were inspected daily and were provided food and water ad libitum. Mice were housed by subgroup in filtertop Macrolon III cages and accommodated with cage enrichment (Igloo’s and nestlets). If mice would have reached any of the humane endpoints prior to scheduled termination they would have been euthanized. Endpoints were defined as: >20% weight loss, pumping breath, inactive, feeling cold, bulging. None of the mice reached a humane end point during the study, however two animals did not wake up after they were anesthetized during virus infection and one animal was found dead in the cage, likely due to narcosis. When the experimental end point was reached, mice were anesthetized (isoflurane/oxygen) and bled by orbital puncture. Mice were anesthetized during influenza challenges by isoflurane in O2 to minimize suffering.

### Study design

HLA-A2 transgenic female mice, B6.Cg-Immp2I-Tg (HLA-A/H2-D)2Enge/J (Jackson Laboratory, USA) arrived at the Animal Research Centre (Bilthoven, the Netherlands) at least 2 weeks before commencement of the study for acclimatization. Mice were aged between 5 and 10 weeks at day of arrival. The animals were semi-randomly distributed; older mice were used first and younger mice were used in the later experiments.

For the dose finding experiment mice were vaccinated with different doses (10 nm – 30 nm – 90 nm – 270 nm) of the WT or MOD1 peptide (Table 1). Each treatment group consisted of four animals, while both the negative (mock vaccination) and the positive (virus challenge) control group consisted of 8 animals. For practical reasons the experiment was divided into four sub experiments, in which 1 mouse from the peptide vaccination group was vaccinated or sacrificed and 2 mice from the negative and positive control group. Mice were housed together based on sub experiment and based on the peptide with which they were vaccinated (WT or MOD1) with 1 mock-vaccinated mouse in every cage to reduce cage effects. The mice infected with IAV were housed separately from the vaccinated mice. On day 0, groups were vaccinated subcutaneously (s.c.) in the left groin fold. On day 21, mice received the booster vaccination s.c. in the right groin fold. Ten days after the booster vaccination, mice were sacrificed.

For the experiment comparing vaccination with MOD1, MOD2 MOD3 and MOD4, each treatment group consisted of 8 animals, which were either vaccinated with a peptide or infected with influenza virus (PR8 strain, 1 × 103 TCID50 in 50 µl i.n.). The control group consisted of 4 animals and received a mock-vaccination consisting of DMSO. Peptide vaccinations consisted of the WT peptide or one of the 4 CPLs (MOD1, MOD2 MOD3 or MOD4, Table 1). Mice were vaccinated s.c. on day 0, in the left groin fold. At day 21, the mice received their booster vaccination, s.c. in the right groin fold, while the positive control group received a virus infection i.n. with 50 µl PR8 virus (1 × 103 TCID50). For the first part of the experiment, 8 mice per peptide vaccination were euthanized 10 days after the booster vaccination (day 31), as well as 8 mice after the IAV infection, and 4 mice of the mock group. For practical reasons, the experiment was divided into two parallel groups (A and B) with a time difference of 1 week, with 4 mice (treatment group and virus infection) or 2 mice (mock vaccination) per subgroup.

We continued the experiment with MOD1 and MOD2 of the CPLs, to investigate early memory formation and protection against challenge. For this part, extra subgroups of mice were made in the groups receiving vaccination with the WT, MOD1 or MOD2 peptide (total of 16 mice per vaccination type). These mice received their vaccination or challenge at the same time as the mice described above, however, per vaccination, 8 of the 16 mice were sacrificed at day 51 (30 days after the booster vaccination); while the other 8 mice received a virus challenge i.n. with 50 ul PR8 (1×103 TCID50) at day 54 and were sacrificed at day 58 (5 days after the virus challenge). Both section moments were controlled by a mock vaccination (2 mice at day 51 and 8 mice after the virus challenge), and mice immunized by infection with IAV (8 mice at both day 51 and day 58).

### Sample collection

Of every mouse, spleen, heparin blood and serum were collected. Measurements on spleen and blood were performed at the day of withdrawal, serum was stored at −20 °C. Spleens were homogenized and passed through 70 um filters (BD biosciences), washed with RPMI 1640 containing 10% FCS and 100 U/ml penicillin, streptomycin, and glutamate. Erythrocytes were lysed using ACK-buffer (NH_4_Cl 0.15 M, KHCO_3_ 0.01 M, Na_2_EDTA 0.1 mM). At day 59, besides the collection of the spleen, heparin blood and serum, also the lungs were collected. The right part of the lung was collected in formalin and used for histopathology, the left part of the lung was collected in Lysing Matrix A tubes and stored at −80 °C for virus titer analyses.

### Peptide vaccination

Peptides were adjuvanted with Incomplete Freund’s Adjuvant (IFA) (1/1 (V/V)) and CpG (50 µg/mouse) and supplemented with PBS. The mixture was vortexed for 1 h. Mice were vaccinated with the indicated peptides at their respective doses in a volume of 100 µl. Before use, the freeze-dried peptides were dissolved in DMSO, aliquoted and stored at −20 °C. Mock vaccination consisted of DMSO and adjuvants.

CPLs were designed and synthesized at the Department of Cell and Chemical Biology, Leiden University Medical Centre, by standard solid-phase peptide synthesis using Syro I and Syro II synthesizers^30^. Amino acids were purchased from Chiralix, NovaBiochem, Chem-Impex or Creo Salus. Resins were purchased pre-loaded with proteogenic amino acids (Nova Biochem) or loaded with non-proteogenic amino acids. Typically, 2-chlorotrityl chloride resin corresponding to a loading of 0.3 mmol (Nova Biochem) was swollen in dichloromethane (DCM, Biosolve); 0.15 mmol of amino acid and 0.51 mmol di-isopropylethylamine (DIPEA, Sigma-Aldrich) were added and the mixture was shaken for 10 minutes. Another 0.99 mmol DIPEA in DCM was added and the mixture was shaken for one hour. The reaction was quenched by addition of methanol. Peptides were synthesized on a large scale (25–50 μmol) and purified by reversed-phase HPLC (Waters). Masses of all peptides were analyzed by LCMS (Waters) to confirm correct synthesis. For this study, we selected four non-proteogenic peptides, namely [am-phg]ILGFVFTL (d-α-methyl-phenylglycine, MOD1), [3-PYRA]ILGFVFTL (3’-pyridyl-alanine, MOD2), G[NLE]LGFVFT (norleucine L, MOD3) [SOME]ILGFVFTL (O-methyl-L-serine, MOD4), based on their MHC-binding affinity and induced T-cell response^7^. Note that L-amino acids are denoted in uppercase characters and D-amino acids in lowercase characters. WT (M1_58–66)_ and natural variant peptides were synthesized at DGpeptides (China), with a purity of >99%. The WT peptide is homologous to the M1 protein of the Influenza PR8 virus.

### Virus

Influenza A/PR/8/34 (PR8; NIBSC code 16/108) virus was obtained from the National Institute for Biological Standards and Control (NIBSC, Hertfortshire, UK). Influenza viruses were grown on MDCK cells in MEM medium (Gibco; Thermo Fisher Scientific) supplemented with 40 µg/ml gentamicin, 0.01 M Tricine and 2 µg/ml TPCK treated trypsin (all from Sigma-Aldrich). At > 90% cytopathic effect (CPE), the suspension was collected and spun down (4000 x g for 10 minutes) to remove cell debris. Supernatant was collected, aliquoted and frozen at −80 °C.

### ELISpot assay

Pre-coated mouse IFNγ-ELISpot (ALP) plates (Mabtech) were used according to the manufacturer’s protocol. Splenocytes were stimulated with 0.1 nmol/well WT or modified peptide in ELISpot plates at 37 °C. Controls consisted of medium and PMA/ionomycin stimulation. Per well, 400.000 cells were added. After 24 h, the plates were developed according to the manufacturer’s protocol. Plates were dried for 1 night, after which they were analysed using the ImmunoSpot® S6 CORE (CTL, Cleveland, OH). Maximum count was set at 500 spots per well.

### Flowcytometry

Approximately 2 million splenocytes and lysed whole blood cells were stained using the commercial A*0201/ GILG dextramer PE. In the same reaction, samples were also stained with an APC-labeled dextramer, loaded with a modified peptide, corresponding to the CPL vaccination. Surface staining was performed for 30 min at 4 °C with the following antibodies and dilutions: CD3(17A2)-FITC (cat. Nr. 555274, 1:100), CD8a(53–6.7)-PerCP/Cy5.5 (cat. Nr. 551162, 1:100), Fixable Viability Stain 780 (cat. Nr. 565388, 1:2000), CD127(SB/199)-BV421 (cat. Nr. 562959, 1:100), CD62L(MEL-14)-BV786 (cat. Nr. 564109, 1:400), CD44(IM7)-PE/Cy7 (cat. Nr. 560569, 1:800), KLRG1(2F1)-PE/CF594 (cat. Nr. 565393, 1:100), CD4(GK1.5)-BUV395 (cat. Nr. 563790, 1:200) (All BD Biosciences). Data acquisition was performed on an LSRFortessa X-20 and data analysis was performed using FlowJo v10.6.2 (BD) software. We did not detect any dextramer staining on CD4 + T cells.

### Isolation of WT-specific T cells for TCR repertoire analysis

CD8 + T cells were isolated from PBMCs using a negative selection microbeads kit (Miltenyi Biotec) according to the manufacturer’s protocol. CD8 + T cells were subsequently labeled at room temperature for 20 min with the A*0201/GILG dextramer (PE, Immudex) and corresponding dextramers manufactured with the modified peptides (APC, Immudex). Subsequently, surface staining was performed using the following mAbs: CD3(17A2)-FITC (cat. Nr. 555274, diluted 1:100), CD4(GK1.5)-BV510 (cat. Nr. 743155, diluted 1:800), CD8(53–6.7)-BV786 (cat. Nr. 563332, diluted 1:400) (All BD Biosciences). CD3 + CD4 − CD8+dextramer+ cells were then sorted using a FACS Melody (BD) directly into RNAlater (Ambion Inc. Applied Biosystems) and stored at −80 °C for subsequent TCRβ clonotype analysis.

### Preparing TCRβ cDNA libraries for sequencing

mRNA was isolated with the RNA microkit (Qiagen) according to the manufacturer’s protocol. Isolated mRNA was used in the 5’ RACE-based SMARTer Mouse TCR α/β profiling kit (Takara Bio USA, Inc.) to perform sequencing of TCRs, following the manufacturer’s protocol using only the TCRβ-specific primers. Cleanup was performed with AMPURE XP clean-up beads (BD). PCR products were sequenced via Illumina MiSeq paired-end 2 × 300 nucleotide (nt) sequencing.

### TCRβ clonotype analysis

Demultiplexed samples were first merged using tool Paired-End reAd mergeR (PEAR)^31^. flow by aligning the sequences to reference TRBV and TRBJ genes. Clonotypes were defined by their CDR3 amino acid sequence. TCR sequences were only accepted when they consisted of at least 100 sequencing reads, to clean the data from possible errors and contamination. Different cut-offs were tested to make sure the choice of the exact cut-off did not influence our qualitative results. TCR diversity was calculated using the previously described Simpson’s diversity index (Venturi et al. 2007). This index ranges between 0 and 1, with 0 representing minimal diversity and 1 representing maximal diversity.

### Pathology

Pathology scoring of the lung was performed as previously described in ref. ^14^. In brief, after fixation the right half of the lung was embedded in paraffin and sliced into 5 µm thick sections. Haematoxylin and eosin-stained slides were examined microscopically at 5x, 10x and 20x magnification. Pathological scoring distinguished between ‘damage’, ‘peri-inflammation’ and ‘intra-lymphocyte filtrate’. Damage related parameters included hypertrophy, hyperplasia, flattened or pseudo squamous epithelia, necrosis and denudation of bronchi(oli) epithelium, hyperemia of septa and alveolar emphysema and haemorrhages. Inflammation related parameters included (peri)bronchi(oli)tis, interstitial infiltrate, alveolitis and (peri)vasculitis characterized by polymorphonuclear (PMN) cells and macrophages. Intra-lymphocytic infiltration-related parameters included lymphocytes, lymphoblasts, and plasma cells. Pathological findings were scored on a scale of 0 (no aberrations) to 5 (severe damage) of which the median was taken as the ‘end score’ for the damage, peri-inflammation or intra-lymphocyte filtrate for different components (e.g. bronchi, bronchiole, blood vessels, interstitium, alveoli) per mouse. Per mouse, at least 8 microscopic fields were scored. An end score was used to summarize the total pathology of the lung. The percentage affected lung tissue was estimated at 20x magnification. Microscopic slides were randomized and scored blindly.

### Virus titer analysis

Virus titer analysis was performed on tissue of the left half of the lung. For analyses, tissue stored in Lysing Matrix A tubes was thawed and 500 μl of Minimal Essential Media (MEM, Gibco) supplemented with 250 ug/ml gentamicin and TPCK-trypsin was added to each tube. The samples were then dissociated using FastPrep (MP Biomedicals). Samples were spun down for 10 min at 4000 x g and 250 μl of the supernatant was serially diluted and tested in 6-plo on MDCK cells. Cytopathic effect (CPE) was cored after 5 days of culturing and TCID_50_ values were calculated using the Reed & Muench method^32,33^.

### Statistical analysis

Differences between the groups were assessed by first using a One-Way ANOVA (or a Kruskal-Wallis, when data was non-parametric). If the One-Way ANOVA was significant, a (post hoc) Mann-Whitney U tests was performed to compare the groups. For all analyses, *p*-values < 0.05 were considered statistically significant. Data were analyzed using GraphPad Prism 8.3 and SPSS statistics 22 for Windows (SPSS Inc., Chicago, IL, USA).

### Reporting summary

Further information on research design is available in the Nature Research Reporting Summary linked to this article.

### Supplementary information

Supplemental Figures

REPORTING SUMMARY

## Data Availability

The data presented in this study are available on request from the corresponding author.

## References

[1] Padilla-Quirarte HO (2019). Protective antibodies against influenza proteins. Front. Immunol..

[2] Wang Z (2015). Recovery from severe H7N9 disease is associated with diverse response mechanisms dominated by CD8(+) T cells. Nat. Commun..

[3] Wilkinson TM (2012). Preexisting influenza-specific CD4+ T cells correlate with disease protection against influenza challenge in humans. Nat. Med..

[4] Sridhar S (2013). Cellular immune correlates of protection against symptomatic pandemic influenza. Nat. Med..

[5] Rosendahl Huber SK (2015). Synthetic long peptide influenza vaccine containing conserved T and B cell epitopes reduces viral load in lungs of mice and ferrets. PLoS One.

[6] Stephens AJ, Burgess-Brown NA, Jiang S (2021). Beyond just peptide antigens: The complex world of peptide-based cancer vaccines. Front. Immunol..

[7] Rosendahl Huber SK (2016). Chemical modification of influenza CD8+ T-cell epitopes enhances their immunogenicity regardless of immunodominance. PLoS One.

[8] Gianfrani C, Oseroff C, Sidney J, Chesnut RW, Sette A (2000). Human memory CTL response specific for influenza A virus is broad and multispecific. Hum. Immunol..

[9] Eickhoff CS (2019). Highly conserved influenza T cell epitopes induce broadly protective immunity. Vaccine.

[10] UniProt C (2021). UniProt: the universal protein knowledgebase in 2021. Nucleic Acids Res..

[11] Venturi V (2007). Methods for comparing the diversity of samples of the T cell receptor repertoire. J. Immunol. Methods.

[12] Denkberg G, Klechevsky E, Reiter Y (2002). Modification of a tumor-derived peptide at an HLA-A2 anchor residue can alter the conformation of the MHC-peptide complex: probing with TCR-like recombinant antibodies. J. Immunol..

[13] Holland CJ (2018). In Silico and structural analyses demonstrate that intrinsic protein motions guide T cell receptor complementarity determining region loop flexibility. Front. Immunol..

[14] de Jonge J (2016). H7N9 live attenuated influenza vaccine is highly immunogenic, prevents virus replication, and protects against severe bronchopneumonia in ferrets. Mol. Ther..

[15] Berkhoff EG (2006). Fitness costs limit escape from cytotoxic T lymphocytes by influenza A viruses. Vaccine.

[16] Tan AC (2013). The design and proof of concept for a CD8(+) T cell-based vaccine inducing cross-subtype protection against influenza A virus. Immunol. Cell Biol..

[17] Tan AC (2011). Precursor frequency and competition dictate the HLA-A2-restricted CD8+ T cell responses to influenza A infection and vaccination in HLA-A2.1 transgenic mice. J. Immunol..

[18] Sharma AK (2001). Class I major histocompatibility complex anchor substitutions alter the conformation of T cell receptor contacts. J. Biol. Chem..

[19] Stewart-Jones GB, McMichael AJ, Bell JI, Stuart DI, Jones EY (2003). A structural basis for immunodominant human T cell receptor recognition. Nat. Immunol..

[20] Davis MM (2003). The problem of plain vanilla peptides. Nat. Immunol..

[21] Moss PA (1991). Extensive conservation of alpha and beta chains of the human T-cell antigen receptor recognizing HLA-A2 and influenza A matrix peptide. Proc. Natl Acad. Sci. USA.

[22] Nguyen THO (2017). Perturbed CD8+ T cell immunity across universal influenza epitopes in the elderly. J. Leukoc. Biol..

[23] Gil A (2015). Narrowing of human influenza A virus-specific T cell receptor alpha and beta repertoires with increasing age. J. Virol..

[24] Man S (2021). Synthetic peptides with inadvertent chemical modifications can activate potentially autoreactive T cells. J. Immunol..

[25] Ekeruche-Makinde J (2012). T-cell receptor-optimized peptide skewing of the T-cell repertoire can enhance antigen targeting. J. Biol. Chem..

[26] Cornberg M (2006). Narrowed TCR repertoire and viral escape as a consequence of heterologous immunity. J. Clin. Invest..

[27] Davenport MP, Price DA, McMichael AJ (2007). The T cell repertoire in infection and vaccination: implications for control of persistent viruses. Curr. Opin. Immunol..

[28] Simonsa BC, Kalamsa SA (2007). The potential role of epitope-specific T-cell receptor diversity in the control of HIV replication. Curr Opin. HIV AIDS.

[29] Valkenburg SA (2016). Molecular basis for universal HLA-A*0201–restricted CD8+ T-cell immunity against influenza viruses. PNAS.

[30] Hoppes R (2014). Altered peptide ligands revisited: vaccine design through chemically modified HLA-A2-restricted T cell epitopes. J. Immunol..

[31] Zhang J (2014). PEAR: a fast and accurate Illumina Paired-End reAd mergeR. Bioinformatics.

[32] Gerritsen B (2016). RTCR: a pipeline for complete and accurate recovery of T cell repertoires from high throughput sequencing data. Bioinformatics.

[33] Ramakrishnan MA (2016). Determination of 50% endpoint titer using a simple formula. World J. Virol..

