# Supplementary material for: Modified influenza M158–66 peptide vaccination induces non-relevant T-cells and may enhance pathology after challenge
Source: NPJ Vaccines. 2023 Aug 12;8:116. doi: 10.1038/s41541-023-00705-y (PMC10423225; doi:10.1038/s41541-023-00705-y)
Supplement: Supplementary file 1 — Supplemental Figures [file 41541_2023_705_MOESM1_ESM.pdf]

Sup. Fig. 1

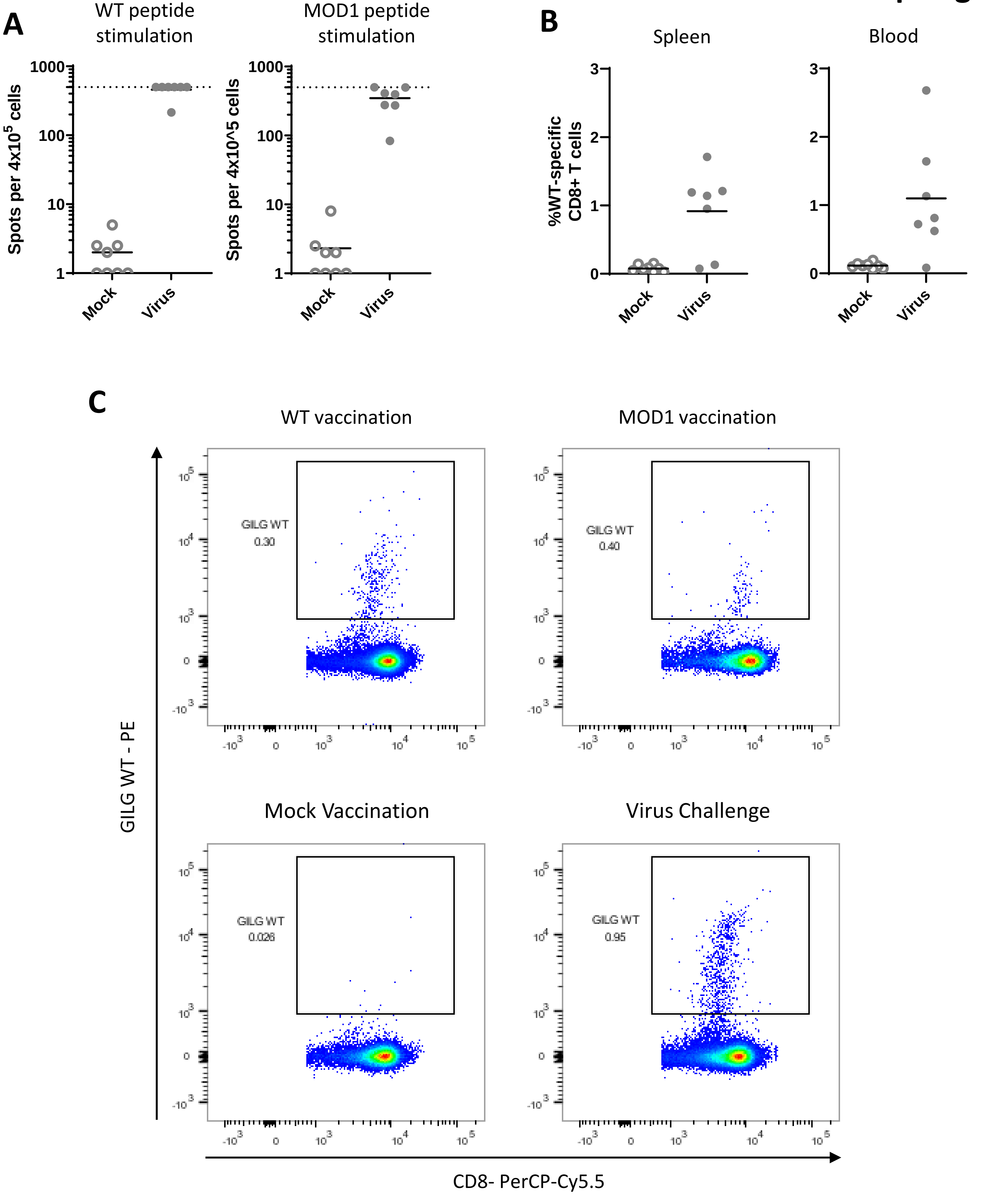

Sup Fig 1: Positive and negative controls of the ELISpot assay and dextramer-staining.
A) For each mock-vaccinated (negative control) and virus-infected (positive control) mouse, $4x10^5$ splenocytes were stimulated with WT peptide or MOD1 peptide. The horizontal dotted line depicts the upper limit of detection (500 spots). B, C) WT-specific CD8+ T-cell frequencies were measured by flow cytometry using WT-peptide loaded dextramers. B) WT-specific CD8+ T cell frequencies in mock-vaccinated and virus-infected mice measured in both spleen and blood. C) Gating of spleen-derived WT-specific CD8+ T cells in mice vaccinated with 10 nmol of WT or MOD1 peptide and the negative (mock vaccinated mice) and positive control (IAV-infection) as measured by flow cytometry. In panels A-B, results of individual mice are shown (points) with the group median (line). Mice vaccinated with mock are shown as open circles and mice infected with influenza a virus are shown as closed circles.

Sup. Fig. 2

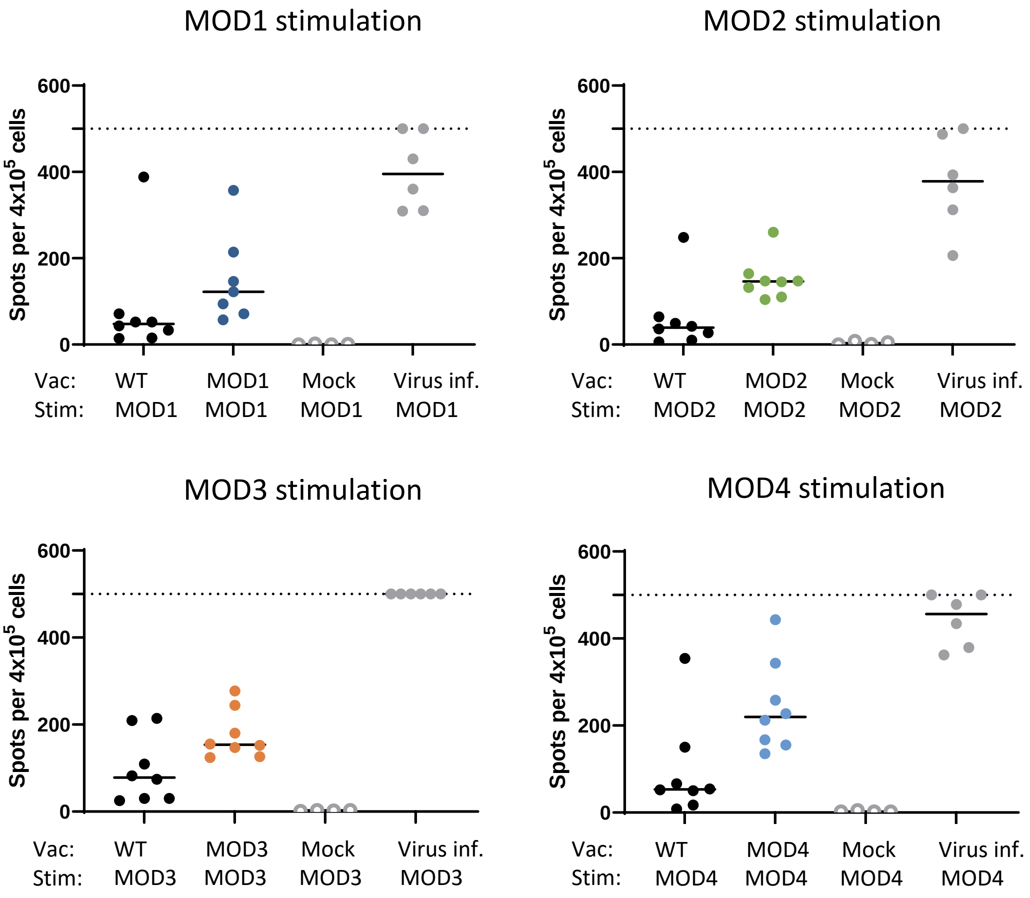

Sup Fig 2: CPLs can stimulate GILG WT-specific memory cells after WT-peptide vaccination and influenza virus infection.

Cellular responses were measured by IFNγ-ELISpot assay after restimulation of 4x10^5 splenocytes with one of the 4 CPLs (MOD1-4) in mice vaccinated with the WT-peptide, the homologous-peptide (MOD1-4), mock or virus-infection. The horizontal dotted line depicts the upper limit of detection (500 spots). Mice vaccinated with WT are depicted in black and mice vaccinated with CPL are indicated by the following colors: MOD1 – dark blue, MOD2 – green, MOD3 – orange, MOD4 – light blue. Mice vaccinated with mock are shown as grey colored open circles.

Sup. Fig. 3

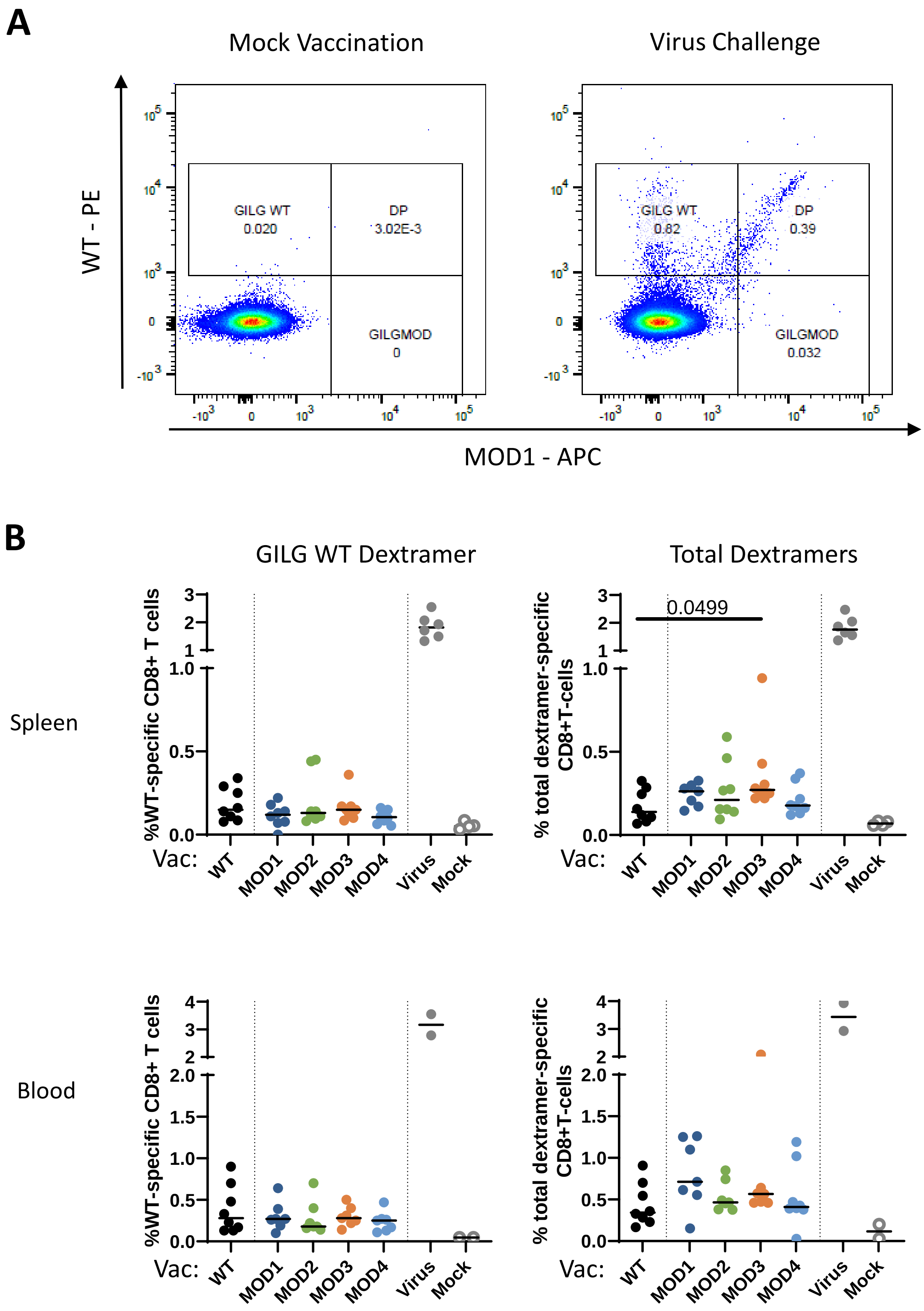

Sup. Fig. 3: Control dextramer-staining and dextramer+ frequencies after CPL vaccination.
A) Example of a dextramer straining on lymphocytes from mock-vaccinated (negative control) and virus-infected (positive control) mice. Lymphocytes were stained with two different dextramers, one loaded with WT and one loaded with MOD1. B) CD8+ T-cell frequencies recognizing WT only (WT+, left) or the sum of the total dextramer+ response (right) as measured in both spleen and blood. Results of individual mice are shown (points) with the group median (line). Mice vaccinated with WT peptide are depicted in black and mice vaccinated with CPL are indicated by the following colors: MOD1 – dark blue, MOD2 – green, MOD3 – orange, MOD4 – light blue. Mice vaccinated with mock are shown as open circles, mice infected with influenza a virus are shown as closed circles, both in grey. Differences between groups were tested for significance by one-way ANOVA, followed by a post-hoc Mann-Whitney U test. Only significant p-values or p-values depicting a trend are depicted in the graphs.

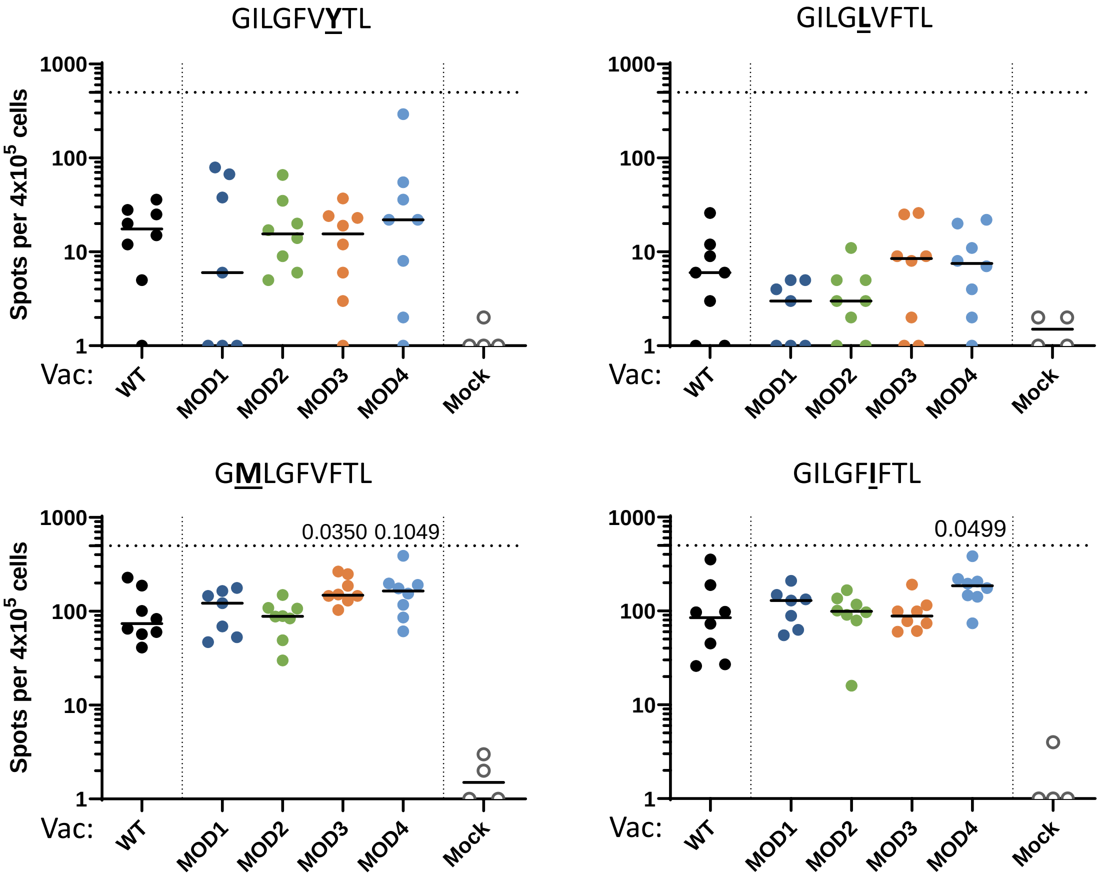

Sup. Fig. 4. IFNγ-response against natural variants of the GILG epitope after CPL vaccination.
IFNγ-responses after stimulation of $4x10^5$ splenocytes with natural variants of the GILG epitope, as measured by IFNγ-ELISpot. Naturally occurring mutations in the GILGFVFTL epitope are indicated in bold and underlined. Results of individual mice are depicted (points) with the group median (line). Mice vaccinated with WT peptide are depicted in black and mice vaccinated with CPL are indicated by the following colors: MOD1 – dark blue, MOD2 – green, MOD3 – orange, MOD4 – light blue. Mice vaccinated with mock are shown as grey colored open circles. Differences between groups were tested for significance by one-way ANOVA, followed by a post-hoc Mann-Whitney U test. Only significant p-values or p-values depicting a trend are depicted in the graphs.

Sup. Fig. 5

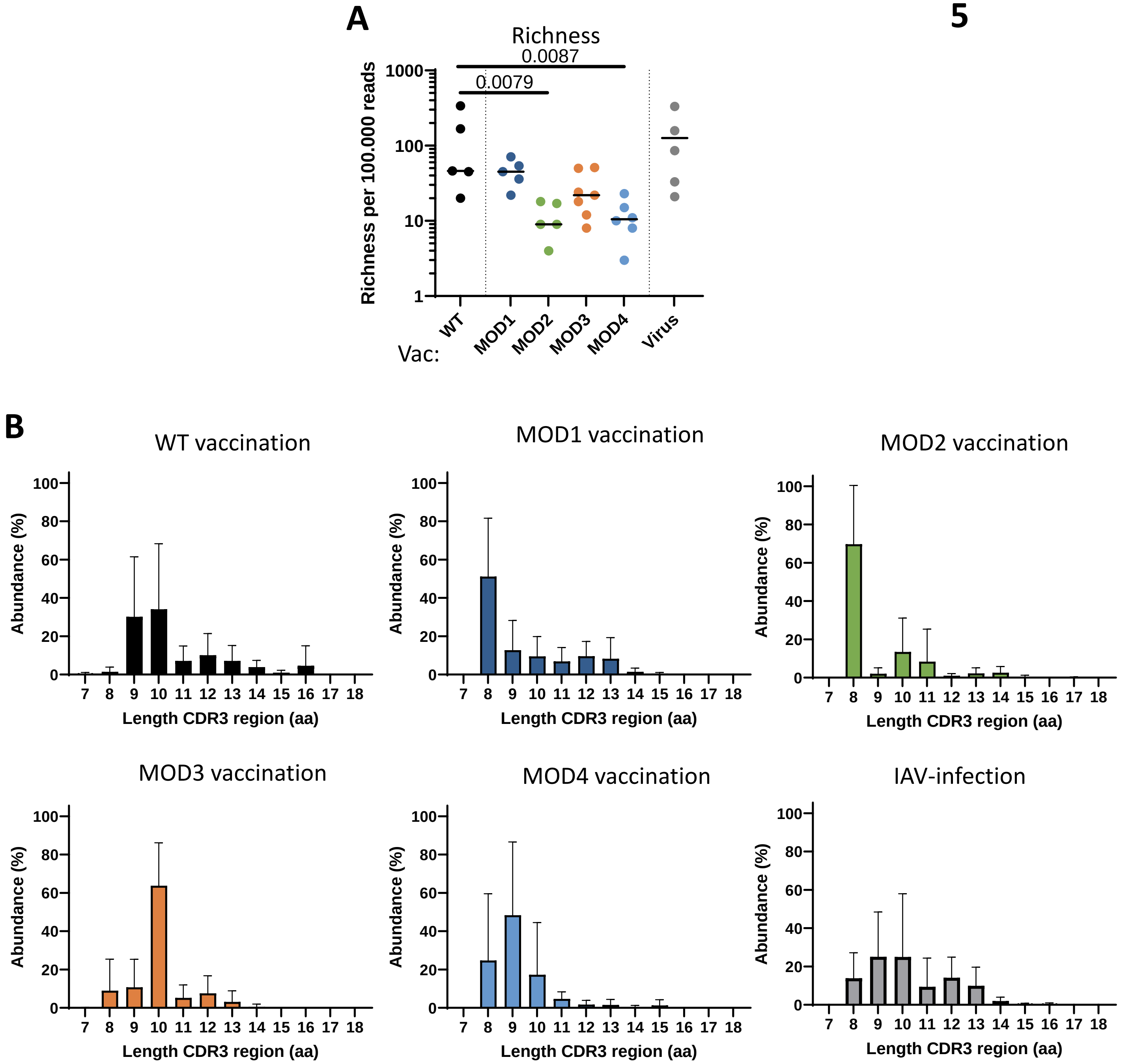

Sup. Fig. 5. Richness and CDR3 length of the WT specific TCR repertoire after peptide vaccination.
A) Richness of the WT-specific T-cell repertoire after vaccination with WT peptide or CPLs and after influenza infection. Richness was calculated by iteratively sampling (100.000 times), normalized to 100.000 reads per sample. Data of individual mice are shown (points) with median (line). B) Distribution of the CDR3 length (in frequency) of the TCR sequences of the GILG-WT specific T-cell repertoire in the different treatment groups. Mice vaccinated with GILG WT are depicted in black and mice vaccinated with CPL are indicated by the following colors: MOD1 – dark blue, MOD2 – green, MOD3 – orange, MOD4 – light blue. Mice infected with influenza a virus are shown as grey colored closed circles. Differences between responses to WT and CPLs are tested using Mann-Whitney U test. P-values are only shown when there was a significant difference between WT and a MOD vaccination.

Sup. Fig. 6

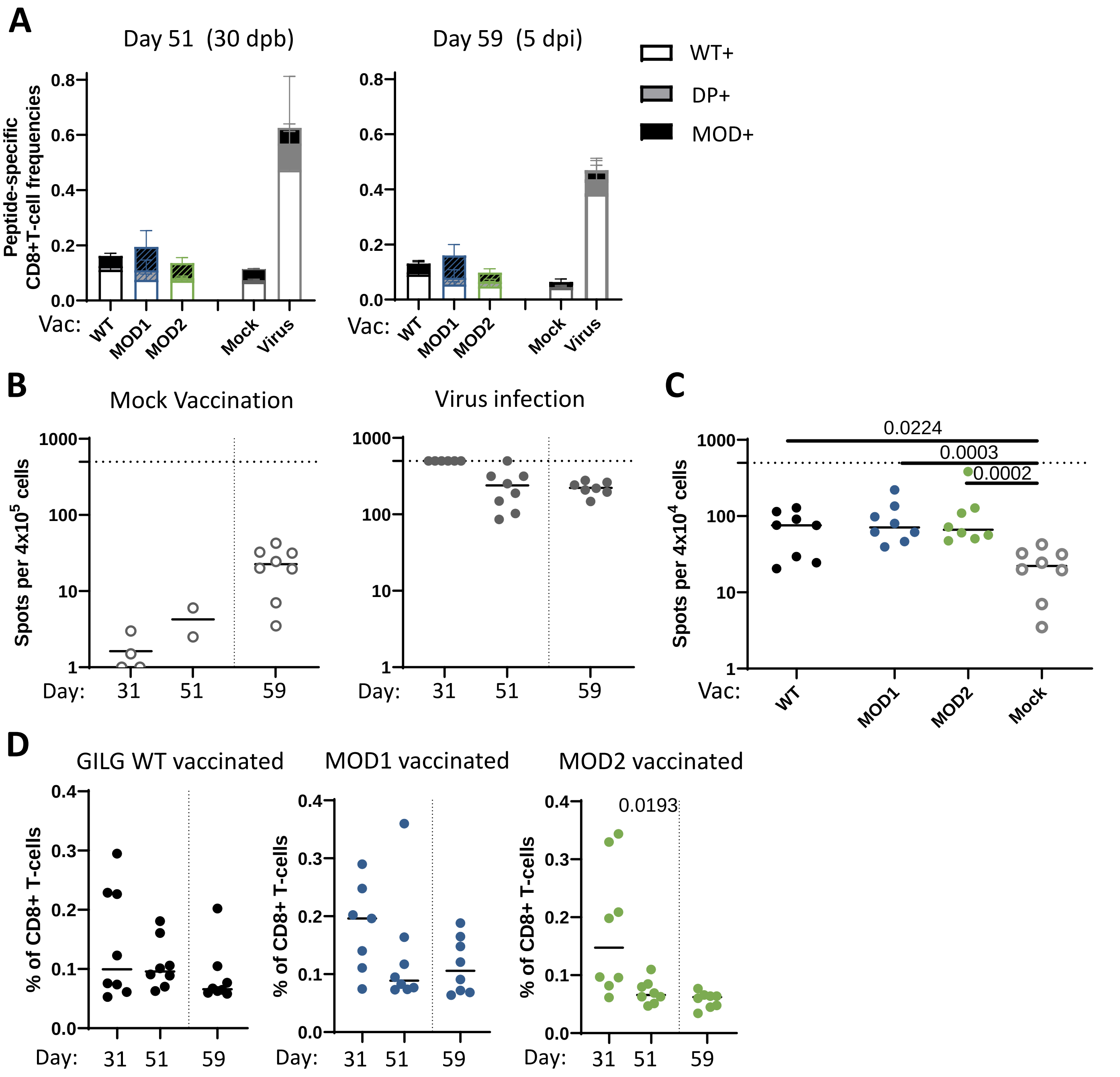

Sup Fig. 6: T-cell memory response after peptide vaccination.
A) Overview of dextramer+ T cells present in the WT+, DP+ and MOD+ gate, mean percentage per group is shown. B, C) Cellular responses measured by IFNγ -ELISpot. B) Responses against WT-peptide in mock vaccinated mice (negative control) and after virus challenge (positive control). C) Comparison of mice vaccinated with WT, MOD1 or MOD2 peptide with mock vaccinated mice after stimulation of splenocytes at day 59. D) Frequency of WT-specific CD8+ T cells within the CD8+ T-cell population of mice vaccinated with WT, MOD1 or MOD2 at different days post vaccination and after IAV-challenge, measured by dextramer straining. In panels B-D, results of individual mice are shown (points) with the group median (line). Mice vaccinated with WT peptide are depicted in black. Mice vaccinated with CPL are indicated by the following colors: MOD1 – dark blue, MOD2 – green. Mice vaccinated with mock are shown as open circles, mice infected with influenza a virus are shown as closed circles, both in grey. Differences between responses to WT and CPLs are tested using Mann-Whitney U test. P-values are only shown when there was a significant difference between WT and a MOD vaccination.

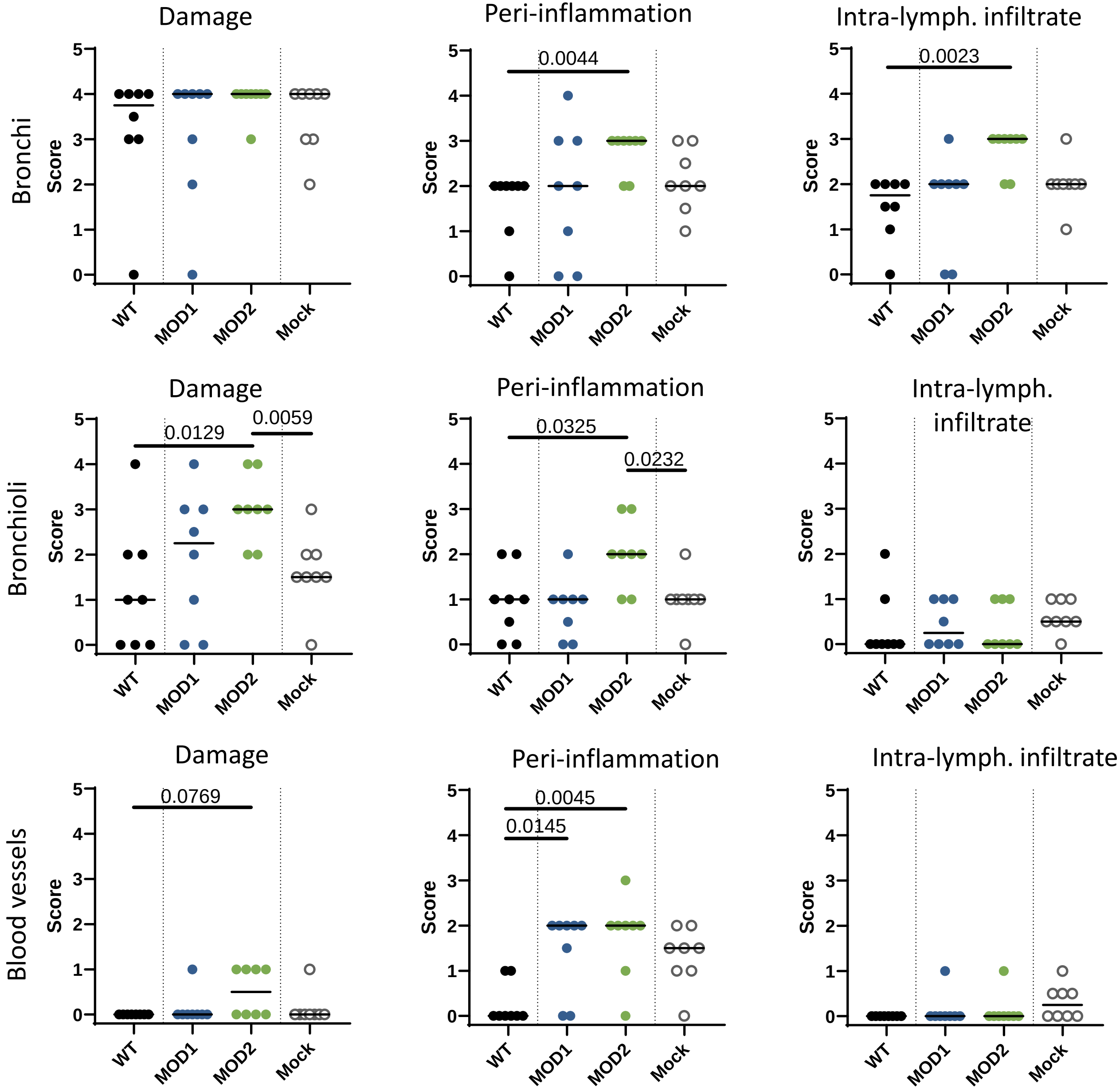

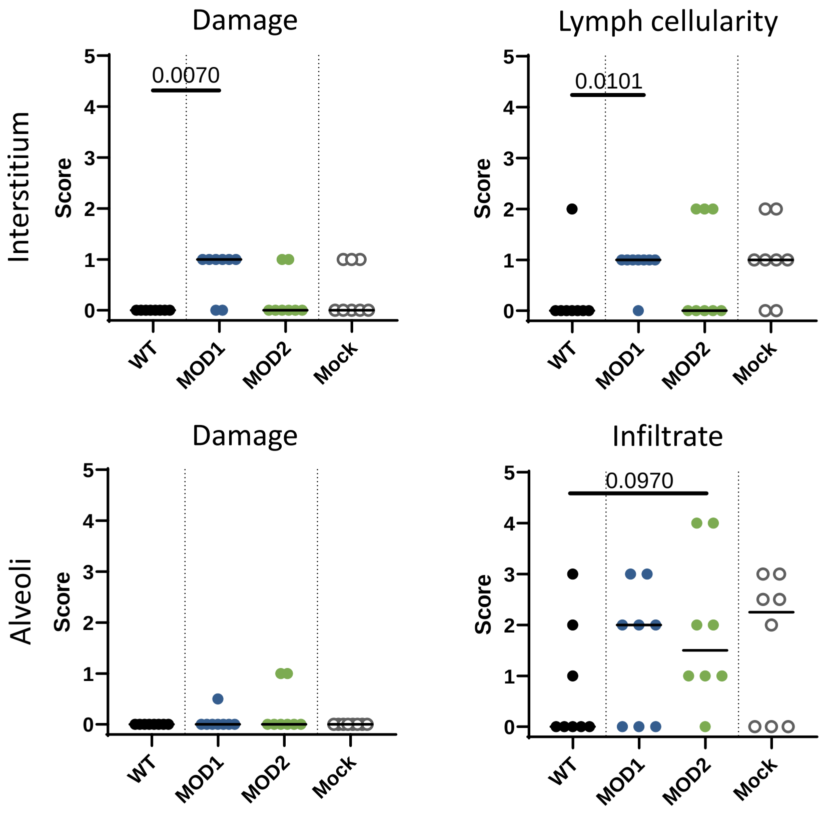

Sup. Fig. 7. Histopathology scores in different parts of the lungs after IAV-challenge in peptide-vaccinated mice.
Overview of the different histopathology scores in bronchi, bronchiole, blood vessels, interstitium and alveoli. Pathology was scored on a scale from 0 (no aberrations) to 5 (severe damage). Per mouse at least 8 microscopic fields were scored; median scores per mouse were plotted. Results of individual mice are shown (points) with the group median (line). Mice vaccinated with WT peptide are depicted in black. Mice vaccinated with CPL are indicated by the following colors: MOD1 – dark blue, MOD2 – green. Mock vaccinated mice are depicted by open grey symbols; IAV-infected mice are depicted by solid grey symbols. Differences between responses to WT and CPLs are tested using Mann-Whitney U test. P-values are only shown when there was a significant difference between WT and a MOD vaccination.
