# Supplementary material for: Modified influenza M158–66 peptide vaccination induces non-relevant T-cells and may enhance pathology after challenge
Source: NPJ Vaccines. 2023 Aug 12;8:116. doi: 10.1038/s41541-023-00705-y (PMC10423225; doi:10.1038/s41541-023-00705-y)
Supplement: Supplementary file 2 — REPORTING SUMMARY [file 41541_2023_705_MOESM2_ESM.pdf]

nature portfolio

Corresponding author(s): Dr Jørgen de Jonge

Last updated by author(s): Aug 1, 2022

Reporting Summary

Nature Portfolio wishes to improve the reproducibility of the work that we publish. This form provides structure for consistency and transparency in reporting. For further information on Nature Portfolio policies, see our Editorial Policies and the Editorial Policy Checklist.

Statistics

For all statistical analyses, confirm that the following items are present in the figure legend, table legend, main text, or Methods section.

n/a	Confirmed	
☐	☒	The exact sample size (n) for each experimental group/condition, given as a discrete number and unit of measurement
☐	☒	A statement on whether measurements were taken from distinct samples or whether the same sample was measured repeatedly
☐	☒	The statistical test(s) used AND whether they are one- or two-sided<br>*Only common tests should be described solely by name; describe more complex techniques in the Methods section.*
☒	☐	A description of all covariates tested
☐	☒	A description of any assumptions or corrections, such as tests of normality and adjustment for multiple comparisons
☐	☒	A full description of the statistical parameters including central tendency (e.g. means) or other basic estimates (e.g. regression coefficient) AND variation (e.g. standard deviation) or associated estimates of uncertainty (e.g. confidence intervals)
☒	☐	For null hypothesis testing, the test statistic (e.g. F, t, r) with confidence intervals, effect sizes, degrees of freedom and P value noted<br>*Give P values as exact values whenever suitable.*
☒	☐	For Bayesian analysis, information on the choice of priors and Markov chain Monte Carlo settings
☒	☐	For hierarchical and complex designs, identification of the appropriate level for tests and full reporting of outcomes
☒	☐	Estimates of effect sizes (e.g. Cohen's d, Pearson's r), indicating how they were calculated

Our web collection on statistics for biologists contains articles on many of the points above.

Software and code

Policy information about availability of computer code

Data collection	Flow cytometry data was acquired on an LSRFortessa X-20
Data analysis	ELISpot data were analysed using the ImmunoSpot® S6 CORE (CTL, Cleveland, OH). Flow cytometry data was analysed using FlowJo v10.6.2 (BD) software. Data were analyzed using GraphPad Prism 8.3 and SPSS statistics 22 for Windows (SPSS Inc., Chicago, IL, USA). Clonotype information was identified from the raw sequence data using the pipeline RTCR [32]

For manuscripts utilizing custom algorithms or software that are central to the research but not yet described in published literature, software must be made available to editors and reviewers. We strongly encourage code deposition in a community repository (e.g. GitHub). See the Nature Portfolio guidelines for submitting code & software for further information.

Data

Policy information about availability of data

All manuscripts must include a data availability statement. This statement should provide the following information, where applicable:

- Accession codes, unique identifiers, or web links for publicly available datasets
- A description of any restrictions on data availability
- For clinical datasets or third party data, please ensure that the statement adheres to our policy

The data presented in this study are available on request from the corresponding author.

Human research participants

Policy information about studies involving human research participants and Sex and Gender in Research.

Reporting on sex and gender	No human derived data was used for this study.
Population characteristics	See above.
Recruitment	See above.
Ethics oversight	See above.

Note that full information on the approval of the study protocol must also be provided in the manuscript.

Field-specific reporting

Please select the one below that is the best fit for your research. If you are not sure, read the appropriate sections before making your selection.

☒ Life sciences ☐ Behavioural & social sciences ☐ Ecological, evolutionary & environmental sciences

For a reference copy of the document with all sections, see nature.com/documents/nr-reporting-summary-flat.pdf

Life sciences study design

All studies must disclose on these points even when the disclosure is negative.

Sample size	Sample size was determined based on the difference in dextramer frequencies we wanted to obtain between the WT-peptide vaccinated mice and the mod vaccinated mice.
Data exclusions	Only datapoint that was excluded in the flow cytometry data was 1 mouse of the MOD1 group (Fig 2+3) as the cells died during isolation and could not be used for measurements. Also datapoints in the ratio calculations were excluded, when there were less than 40 cells to do a calculation, to make sure the ratios would not be misleading.
Replication	The experiment was split into two sub-experiments that were carried out separately (1 week time difference, n = 4 per group), but analyzed together (for a total of n = 8).
Randomization	The animals were semi-randomly distributed; older mice were used first and younger mice were used in the later experiments.
Blinding	The investigators were not blinded to the study as they had to prepare the vaccine and (mock-)infection inoculum and supervise the correct injection of the inoculum into the mice.

Reporting for specific materials, systems and methods

We require information from authors about some types of materials, experimental systems and methods used in many studies. Here, indicate whether each material, system or method listed is relevant to your study. If you are not sure if a list item applies to your research, read the appropriate section before selecting a response.

Materials & experimental systems

n/a	Involved in the study	
☐	☒	Antibodies
☒	☐	Eukaryotic cell lines
☒	☐	Palaeontology and archaeology
☐	☒	Animals and other organisms
☒	☐	Clinical data
☒	☐	Dual use research of concern

Methods

n/a	Involved in the study	
☒	☐	ChIP-seq
☐	☒	Flow cytometry
☒	☐	MRI-based neuroimaging

Antibodies

Antibodies used	CD3(17A2)-FITC, CD8a-PerCP/Cy5.5 (53-6.7), Fixable Viability Stain 780, CD127(SB/199)-BV421, CD62L(MEL-14)-BV786, CD44(IM7)-PE/Cy7, KLRG1(2F1)-PE/CF594, CD4(GK1.5)-BUV395 (All BD Biosciences).

Validation	*Describe the validation of each primary antibody for the species and application, noting any validation statements on the manufacturer's website, relevant citations, antibody profiles in online databases, or data provided in the manuscript.*

Animals and other research organisms

Policy information about [studies involving animals](); [ARRIVE guidelines]() recommended for reporting animal research, and [Sex and Gender in Research]()

Laboratory animals	HLA-A2 transgenic female mice, B6.Cg-Immp2l-Tg (HLA-A/H2-D)2Enge/J (Jackson Laboratory, USA)
Wild animals	study did not involve wild animals
Reporting on sex	Only female mice were used for this study, as it is known that male and female mice react differently to influenza infection, which would mean we would have needed double the amount of mice when using both sexes. Karnam et al. 2012
Field-collected samples	Study did not involve samples collected from the field.
Ethics oversight	The study was approved by the Animal Welfare Body of Poonawalla Science Park, Animal Research Center (Bilthoven, The Netherlands) under permit number AVD3260020173890 of the Dutch Central Committee for Animal experiments.

Note that full information on the approval of the study protocol must also be provided in the manuscript.

Flow Cytometry

Plots

Confirm that:

☒ The axis labels state the marker and fluorochrome used (e.g. CD4-FITC).

☒ The axis scales are clearly visible. Include numbers along axes only for bottom left plot of group (a 'group' is an analysis of identical markers).

☒ All plots are contour plots with outliers or pseudocolor plots.

☒ A numerical value for number of cells or percentage (with statistics) is provided.

Methodology

Sample preparation	Approximately 2 million splenocytes and lysed whole blood cells were stained with an dextramers, next surface staining was performed for 30 min at 4 °C
Instrument	Data acquisition was performed on an LSRFortessa X-20.
Software	Data analysis was performed using FlowJo v10.6.2 (BD) software.
Cell population abundance	*Describe the abundance of the relevant cell populations within post-sort fractions, providing details on the purity of the samples and how it was determined.*
Gating strategy	Lymphocytes (FSC-A/SSC-A) - single cells (FSC-A/FSC-H) - Live Dead (Livedead/SSC-A)- CD3+ (CD3/SSC-A)- CD8+ (CD8a/CD4) - GILG+, MOD+ DP+ (GILGmod/GILGWT dextramer).

☐ Tick this box to confirm that a figure exemplifying the gating strategy is provided in the Supplementary Information.
